# Care strategies for children with tracheostomy at school: a scoping review

**DOI:** 10.1590/1980-220X-REEUSP-2025-0096en

**Published:** 2025-11-03

**Authors:** Fernanda Borges Pessanha, Jéssica Renata Bastos Depianti, Thaís Guilherme Pereira Pinheiro Pimentel, Ivone Evangelista Cabral

**Affiliations:** 1 Universidade Federal do Rio de Janeiro, Escola de Enfermagem Anna Nery, Rio de Janeiro, RJ, Brazil.; 2 Universidade do Estado do Rio de Janeiro, Faculdade de Enfermagem, Rio de Janeiro, RJ, Brazil.

**Keywords:** Child Health, Tracheostomy, Mainstreaming, Education, School Nursing, School Health Services

## Abstract

**Objective:**

To map care strategies for children with tracheostomies in school settings.

**Method:**

A scoping review was conducted following the JBI® methodology. A comprehensive search strategy was employed across health, education, and gray literature databases in July 2024. The included studies described guidelines, care policies, and the experiences of family members, healthcare professionals, and education professionals regarding the care of children (6–12 years old) with tracheostomies who regularly attend school.

**Results:**

The 30 texts retrieved that met the eligibility criteria identified three models of school healthcare: family-based, health-education intersectoral, and school health outpatient-based.

**Conclusion:**

The successful inclusion of children with tracheostomies in educational settings necessitates intersectoral health-education coordination in the development of guidelines. Review protocol registered at https://osf.io/3tcg4.

## INTRODUCTION

The Brazilian Academy of Pediatric Otorhinolaryngology’s first consensus on the clinical management of children with tracheostomy (CWT) after hospital discharge emphasizes the gap in organized data on these patients, the necessity for supplies to ensure ongoing care, and the importance of training healthcare professionals and laypeople to monitor their condition. These children remain connected to the tertiary healthcare system^(1,2)^. Additionally, there is limited social recognition that they belong to a group requiring specific care for their post-hospital life^(3)^. Even after discharge, they continue to need continuous and complex care, classifying them as a subgroup of children with special health needs (CSHCN)^(2,3)^.

CWT is among the devices that elicit the greatest concern regarding safety and the well-being of children^(4,5)^. Moreover, reliance on this device may present challenges to the social integration of children with special healthcare needs (CSHCN), as they require complex and continuous care to maintain a patent airway. This underscores the importance of delivering these services in a setting that offers logistical advantages, such as access to electrical outlets and a reliable power supply network^(5)^. In educational environments, vulnerability is increased due to the challenges associated with guaranteeing safe school transportation, the limited availability of personnel capable of responding to respiratory emergencies, and the complexities involved in upholding these measures care^(5)^. As families transition into the school age, they express concerns regarding school inclusion amid circumstances of inadequate preparation to accommodate them safely^(3,6)^.

The Brazilian National Education Guidelines and Bases Law (Law 9,394/1996) assures that children’s education shall not be disrupted under any circumstances, whether at home or in a hospital setting. When at home, homeschooling is designated for children who are unable to attend traditional schools due to mobility impairments or other reasons homeschooling^(7)^.

In recent years, the issue of school inclusion in the United States, England, and Japan has been addressed through inclusive strategies that facilitate continuity of care^(8, 9, 10)^. In these countries, rights to social inclusion, peer interaction, and engagement with the school community are protected. Consequently, there is a need for a thorough mapping of this evidence, as current policies and programs in Brazil are inadequate to address the complexities of ongoing care for children with CWT school^(1)^. The School Health Program (referred to in Brazilian Portuguese as Programa Saúde na Escola - PSE), an intersectoral health and education initiative, possesses a consultative nature, characterized by scheduled visits conducted by professionals from the Family Health Strategy school^(6)^.

International evidence demonstrates that children with CWT are able to attend mainstream educational institutions, provided that the institution is sufficiently equipped to deliver the requisite care, including suctioning and cannula clearance necessary^(8, 9, 10)^. There is currently a lack of synthesized evidence in systematic reviews regarding care strategies that support the school inclusion of CSHCN with CWT. Consequently, there is a need to identify and categorize these strategies. An initial search of PROSPERO, PubMed, Cochrane Database of Systematic Reviews, and JBI Evidence Synthesis revealed no registered, ongoing, or completed reviews on this topic. Thus, this study aims to map the care strategies for children with CWT in school environments.

## METHOD

### Study Design

**Figure 1:**
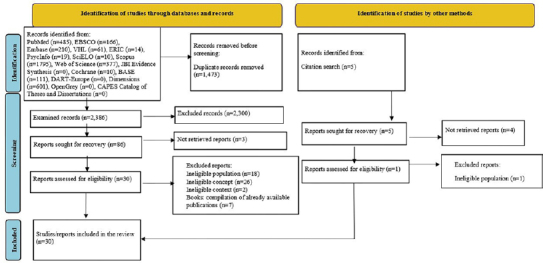
Preferred Reporting Items for Systematic Reviews and Meta-Analyses extension for Scoping Reviews flowchart applied to the registration of the selection of publications subject to analysis in this scoping review^(15)^, 2024.

This scoping review was conducted in July 2024 (the last date of application of the search strategy in each database was the 24^th^) according to JBI^®^ methodology for scoping reviews^(11)^, and the report followed Preferred Reporting Items for Systematic Reviews and Meta-Analyses extension for Scoping Reviews (PRISMA-ScR) transparency guidelines^(12)^ (see Figure 1). The protocol for this scoping review was registered, *a priori*, on the Open Science Framework platform (https://osf.io/ekp7u/), with the identifier DOI 10.17605/OSF.IO/EKP7U.

### Review Question

To fulfill the review objective, the mnemonic PCC was used to develop the research question: P stands for participant or population (healthcare and education professionals, school administrators, and family members); C signifies concept (care strategies for enrolling and retaining children with TCT); and C represents context (regular schools). Therefore, the question becomes: what care strategies do healthcare and education professionals, school administrators, and family members employ to enroll and retain children with TCT in regular schools?

### Eligibility Criteria

Included were articles involving family caregivers of children aged 6 to 12 with CWT who attended school, as well as general healthcare professionals, school health nurses, education staff, and school personnel who cared for or supported these children. The review also incorporated studies focusing on care strategies to promote the enrollment and ongoing attendance of these children in various school settings—such as classrooms, playgrounds, sports courts, and transportation. Additionally, studies concerning the organization of healthcare services, including school health clinics and school healthcare programs, were considered. Regarding the study design, scientific articles encompassing quantitative, qualitative, and mixed methods approaches, as well as case studies related to school healthcare services for children with CWT, were incorporated. A comprehensive search was additionally conducted, including gray literature such as these, dissertations, care programs, guidelines, and editorials. No restrictions were imposed based on date or language during the search.

### Search Strategy and Study Selection

In the JBI® methodology, the search strategy is executed in three distinct stages: 1) a preliminary search is conducted on the PubMed and CINAHL databases to identify the most prevalent terms within the titles and abstracts of relevant articles pertaining to the review question; 2) indexed terms are employed to assist in the development and refinement of a sensitive and specific search strategy; 3) the complete search strategy is implemented, incorporating Health Sciences Descriptors/Keywords, Medical Subject Headings, their respective synonyms, keywords, and entry terms. This final strategy is tailored for each information source or database. Chart 1 illustrates the search strategy applied to the PubMed repository.

**Chart 1:** Example of a search strategy applied to the PubMed library (MEDLINE).

Source of information	Search strategy on July 24, 2024
PubMed	((Tracheostomy[mh] OR Tracheostom*[tiab]) AND (“Infant”[mh] OR infant*[tiab] OR Child[mh] OR child*[tiab] OR Children[tiab] OR “Disabled Children”[tiab] OR “Children with Disabilities”[tiab] OR “Children with Disability”[tiab] OR “Disabled Child”[tiab] OR “Handicapped Children”[tiab] OR children special health care need*[tiab] OR “exceptional child”[tiab] OR “exceptional children”[tiab] OR “technology dependent child”[tiab] OR “technology dependent children”[tiab] OR “Special Needs Child”[tiab] OR “Special Needs Children”[tiab])) AND (“Mainstreaming, Education”[mh] OR Education Mainstreaming[tiab] OR Educational Mainstreaming[tiab] OR “Return to School”[mh] OR Return School*[tiab] OR “School Health Services”[mh] OR “School Health Services”[tiab] OR “School Based Health Services”[tiab] OR “School Based Services”[tiab] OR “School Health Promotion”[tiab] OR “School Health Service”[tiab] OR “School Based Health Service”[tiab] OR “School Based Health Services”[tiab] OR “School Based Service”[tiab] OR “School Based Services”[tiab] OR Middle School Teacher*[tiab] OR Elementary School Teacher*[tiab] OR “educational institution”[tiab] OR “school building”[tiab] OR “school organisation”[tiab] OR “school organization”[tiab] OR School*[tiab] OR “Elementary Education”[tiab] OR “Elementary School”[tiab] OR “Middle Schools”[tiab] OR “Primary Education”[tiab] OR “Secondary Education”[tiab] OR “intermediate school”[tiab] OR “junior high school”[tiab] OR “junior secondary school”[tiab] OR “lower secondary school”[tiab] OR “Schools”[mh] OR Primary School* OR Secondary School*[tiab] OR “Schools, Nursery”[mh] OR Nursery School*[tiab] OR “Students”[mh] OR Student*[tiab] OR “Education, Special”[mh] OR Special Education*[tiab] OR “Education”[mh] OR Education[tiab] OR Educational Activit*[tiab] OR Literacy Program*[tiab] OR “Teaching”[majr] OR Teaching[tiab] OR Educational[tiab] OR “Schools, Nursing”[mh] OR Nursing School*[tiab] OR Nurse Training School*[tiab] OR school aged[tiab])

The reference lists of all included sources of evidence were scrutinized to identify additional studies. There were no restrictions concerning language or time frame. The most recent search was executed on July 24, 2024. The databases queried encompassed PubMed, CINAHL (EBSCOhost), ERIC, PsycInfo, Web of Science Core Collection, Scopus/CAPES Journal Portal, SciELO, LILACS/Virtual Health Library, Cochrane Database of Systematic Reviews, and JBI Evidence Synthesis. The search for unpublished studies and gray literature incorporated OpenGrey, CAPES Dissertation and Theses Catalog, BASE, DART-Europe, Dimensions Platform, as well as manual examinations of reference lists from the included full-text articles. *Data extraction and analysis*

To select the sources of evidence, the identified records were collated and uploaded to Mendeley™, with duplicates removed. The entire study selection process was conducted using the Rayyan tool by two independent reviewers, thereby minimizing the risk of bias in the study selection process according to the inclusion criteria for the review^(13)^. Any disagreements that emerged among reviewers were resolved through discussion or by involving a third reviewer.

For data extraction^(14)^, a tool developed by the authors was employed. Two reviewers independently extracted data from the results of the selected studies. They entered those strategies that addressed the enrollment and retention of children with CWT in regular school classes into the tool. Subsequently, the data were presented to a third reviewer, possessing expertise in the methodology and subject matter, who selected those most pertinent to the research question of this scoping review for analysis. In instances of disagreement, the three reviewers convened to reach a consensus.

The main findings are presented using basic descriptive content analysis and organized according to this study’s review questions. The extracted data are shown in tables and diagrams. A narrative summary accompanies the tabulated results^(14)^.

## RESULTS

A total of 30 studies were retained for data extraction and included in the review. The results of the search and study inclusion process are presented in accordance with the PRISMA-ScR 2020 guidelines flowchart^(15)^ (Figure 1).

### Characterization of Included Studies

Of the 30 studies, 24 were from the United States^(8,16, 17, 18, 19, 20, 21, 22, 23, 24, 25, 26, 27, 28, 29, 30, 31, 32, 33, 34, 35, 36, 37, 38)^, three from England^(9,39,40)^, two from Japan^(10,41)^, and one from South Africa^(42)^ (see Chart 2). Seven studies were qualitative^(9,10,16, 17, 18,41,42)^—six with individual interviews^(9,10,16, 17, 18,42)^ and one with a focus group^(41)^; five were quantitative using questionnaires^(34,36, 37, 38,40^); two used mixed methods^(19,35^); and 16 included clinical practice guidelines^(20, 21, 22, 23, 24, 25,32)^, case reports^(26,31)^, consultative models^(8,28,29)^, editorials^(39)^, and non-systematic reviews^(33)^. Quantitative studies involved 956 participants, while qualitative studies included 839 individuals, and two case reports examined seven children. The search covered 1988– 2021, identifying 11 studies before 2000^(8,19,21, 22, 23, 24, 25, 26,29,30,33,35)^ and 19 after 2000^(9–10,16, 17, 18,20,24,27–28,30–31,33,35, 36, 37, 38, 39,41–42)^.

**Chart 2:** Characterization of included studies.

Author, year	Country	Study nature/design	Population/setting	Study objective
Mahomva et al.^(42) 2017^	South Africa	Qualitative	Four mothers, two fathers; four teachers/Home and school	Recommendations for schools to become safe and accessible for CWT.
Kruger et al.^(16) 2009^	USA	Qualitative	13 nurses/School	Considerations Regarding the Challenges and Advantages of Collaboration Among Nurses, Parents, and Staff in Pediatric Care Settings
Casey^(17) 2002^	USA	Qualitative	18 school nurses/School	School nurse responsibilities for medically fragile students.
Mukherjee et al.^(9)^ 2000	England	Qualitative	58 family members, 34 teachers/School	Challenges Faced by Educators in Providing Care for Children.
Rehm and Rohr^(18)^ 2002	USA	Qualitative	11 family members, nine nurses, eight teachers, five school nurses/Home, classroom, nursing office	The measures implemented by families, educators, and healthcare professionals to promote and safeguard the health and safety of children within educational environments.
Shimizu and Katsuda^(10)^ 2015	Japan	Qualitative	11 teachers/School	Educators’ Perspectives on the Responsibilities of School Nurses in Caring for Technology-Dependent Children.
Shimizu and Suzuki^(41)^ 2015	Japan	Qualitative	21 nurses/School, classrooms, care room	The role of nurses caring for technology-dependent children in schools.
Esperat et al.^(19)^ 1999	USA	Mixed method	618 teachers, 43 school nurses/School	The need for education and healthcare services in the care of CWT in schools.
*American Academy Pediatrics: Committee on Injury and Poison Prevention* ^(20)^ 2001	USA	Practice guidelines report	NA	Procedures guideline for CWT during transport.
Graff and Ault^(21)^ 1993	USA	Practice guidelines	NA	Guidelines for staff supporting students with special healthcare needs.
Grundfast et al.^(8)^ 1988	USA	Medical center model program	NA	Integration of CWT into the school system.
Haynie et al.^(22)^ 1989	USA	Care guidelines	NA	Guidelines and useful information for transforming technological advances and social commitment for children.
Janz et al.^(23)^ 1993	USA	Care guidelines	NA	Discussion of the issues of including CSHCN in the regular classroom.
Lehr and Greene^(24)^ 2002	USA	Practice guidelines	NA	Considerations, challenges, and procedures for supporting students with complex healthcare needs in educational settings.
Lehr^(25)^ 1990	USA	Practice guidelines	NA	Educational programs for students with complex healthcare needs.
Levine^(26)^ 1996	USA	Case study	Four male students and two female students/School	Ventilator-dependent students’ experiences.
Makrinioti et al.^(39)^ 2021	England	Editorial	NA	A multidisciplinary strategy for CWT’s school reintegration challenges
Margolis^(27)^ 2002	USA	Legislation and policies	NA	Using school healthcare services laws and policies.
Palfrey et al.^(28)^ 1992	USA	Consultative school model	NA	Skills training planning and procedures.
Parette et al.^(29)^ 1994	USA	Inclusion model	NA	Role of pediatric nurses.
Porter et al.^(30)^ 2013	USA	Emergency preparedness	NA	Emergency care guidelines for students at school.
Raymond^(31)^ 2009	USA	Case study	6-year-old boy/School	Integration process, team roles, and implications for school nursing practice.
Smith and Leatherby^(32)^ 1992	USA	Guidelines	NA	Delivering special healthcare procedures to students at school.
Toothaker and Cook^(33)^ 2018	USA	Non-systematic review	NA	School nurse skills for caring for students with CWT.
Mulligan-Ault et al.^(34)^ 1988	USA	Quantitative	150 teachers/School	The role of school nurses.
Patel et al.^(35)^ 2009	USA	Mixed method	23 children/Hospital	CWT’s school experience.
Pufpaff et al.^(36)^ 2015	USA	Quantitative	76 school nurses/School	Collaboration between school nurse and teacher.
Smith et al.^(40)^ 2003	United Kingdom	Quantitative	11 family members and the child’s caregiver at school/Hospital	Identification of children attending school and the support they receive.
Heller et al.^(37)^ 2000	USA	Quantitative	342 teachers, 49 non-nursing healthcare professionals, 21 nurses, ten school administrators	Health skills training for non-medical staff to ensure safety.
Sapien and Allen^(38)^ 2001	USA	Quantitative	230 school nurses/School	Readying public schools for new health requirements per guidelines.

USA – United States of America. NA – not applicable; CWT – children with tracheostomy; CSHCN – children with special healthcare needs. Source: prepared by the authors.

**Figure 2:**
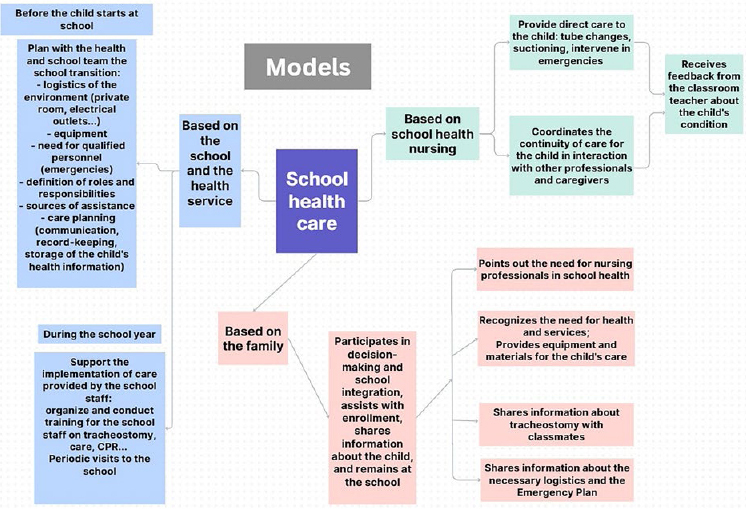
School healthcare models for children with special health needs living with a tracheostomy.

Source: prepared by the authors.

The approaches used by healthcare professionals, education professionals, and family caregivers to enroll and support the ongoing education of children with CWT in regular schools are described in three models, as shown in Figure 2.

### School Healthcare Model Based on a Partnership between School and Healthcare Service

The school-based and healthcare service-based school healthcare model emphasizes a collaborative partnership among the referral service, the school, and community resources for the continuity of care for children. Collectively, they strategize and implement actions to ensure student enrollment and retention. Specifically, this involves implementing recommendations from healthcare professionals to address the particular needs of children within the school environment and providing requisite equipment; identifying emergency situations related to CWT and establishing contact procedures with the emergency team (ambulance, rescue, or emergency department); recruiting qualified personnel for care provision; organizing visits from healthcare services to the school; and scheduling training sessions for school staff conducted by healthcare professionals services^(8,9,18,19,21, 22, 23, 24,26,28,29,32,42)^.

To this end, prior to the commencement of the school year, healthcare professionals are expected to engage in emergency management initiatives at the school and to disseminate information regarding the child to the school community. Emphasis is placed on formulating policies, protocols, and algorithms, with critical information pertaining to the child’s health conditions being anticipated and communicated accordingly. An individual meeting involving the child, parents, and guardians is scheduled at the school^(19,20,22,24, 25, 26,30,32,42)^.

Healthcare services share with the school the specifics of caring for CWT, including what constitutes an emergency and the procedures that should be performed in emergency interventions. The care provided is duly documented in case community healthcare services need to be activated. Based on this information, schools adapt their logistics to meet children’s special health needs, providing space outside the classroom for when they are unwell, such as a private room for procedures, air-conditioned, with temperature control, access to water for cleaning materials and hand washing, storage for equipment and supplies, electrical outlets, and alternative energy sources^(8,9,21, 22, 23, 24,28,30,31)^.

As soon as a child’s transition planning for enrollment was completed and environmental adaptive measures were implemented, strategies for their continued attendance at school began, with an assessment after one month of school inclusion. Some studies recommend, as part of the school inclusion policy for CWT, that safe integration requires a nursing professional to be available at the school whenever a child needs them. To ensure their health and safety, some physicians advocate for seating children near the teachers’ desk, keeping a close eye on their behavior. It is imperative to maintain vigilance and inform parents regarding potential risks of infection or early symptoms, enabling them to keep their children at home or seek medical attention promptly^(9,16, 17, 18,21,42)^.

Schools depend on specialized healthcare professionals to prescribe and follow standardized care guidelines for this child. They also assign a healthcare team liaison who, along with the child’s parents, informs them about health issues and participates in care plan decisions. Additionally, they designate responsibility for purchasing and maintaining equipment, disposing of medical waste, and supplying necessary healthcare materials for the child school^(8,9,20, 21, 22,26,40)^.

To ensure that children remain enrolled in school, the school community team receives healthcare service training, which may be conducted either at the school or at the community health unit. This training is provided by home care professionals, clinicians with pediatric care expertise, individuals knowledgeable about children’s rights legislation, previously trained educators, and family caregivers experienced in specific procedures performed on their own children children^(8,18,21, 22, 23, 24, 25, 26, 27, 28, 29, 30, 31,39–40)^. In this regard, training is provided by certified professionals to educate teachers, administrators, assistant staff, school transportation personnel, coordinators, newly hired school health nurses, and classmates—in other words, all individuals who have regular contact with children. When school personnel receive training, specific procedures for managing tracheostomy in educational environments are implemented safely and accurately. The training is grounded in problem-management competencies and skills and is formalized according to the needs of the school community members and caregivers who provide direct care to children at school. Visual resources (videos), instructional sessions, question-and-answer interactions, additional verbal information from families, problem-solving strategies, and periodic visits with refresher sessions are utilized to offer support and sustain skills. The school and healthcare services document and reinforce training periodically, training at least two or three individuals to ensure ongoing support available^(4,8,10,11,13,14,18,20,25,27,29,31,39,40)^.

In summary, qualitative evidence highlights topics covered in training, such as regulations about who can perform specialized and complex procedures in the classroom, access to community resources, and emergency contact information. This includes basic knowledge of CWT applied to the school setting, emergency planning, signs of distress, and children’s respiratory and overall health to address special health needs.

Quantitative evidence, as shown in Table 1, highlights the importance of cardiopulmonary resuscitation (CPR), first aid, and general care in the context of CWT. Training needs include CWT airway suctioning procedures, device use, and oxygen administration^(8,18, 21, 22, 23, 24, 25,27, 28, 29,31–32,42)^.

**Table 1:** Topics covered in school staff training, under the responsibility of the healthcare service, school health nursing, and the school itself, as a strategy for keeping children with tracheostomies in safe environments school^(34,35,37,38,40)^, 1988–2015.

Topics covered in training	Healthcare professional^(37,38)^(n = 572)			Family member and non-professional caregiver(n = 643)		
	School health nursing professional (n = 1,078)	Physician (n = 88)	Rehabilitation professionals (n = 132)	Healthcare service (n = 159)	Family member (n = 444)	Caregiver (teacher + mediator) (n = 199)
First aid^(37)^		256		–	–	–
Cardiopulmonary resuscitation^(37,38)^		526		–	–	–
Aspiration^(37)^ n = 559	283	24	38	48	113	53
General tracheostomy care^(37)^ n = 539	272	17	30	49	128	43
Inhaler/nebulizer use^(37)^ n = 516	250	23	23	28	127	65
Oxygen administration^(37)^ n = 486	273	24	41	34	76	38

Source: Prepared by the authors.

Table 1 shows that qualified healthcare professionals (such as school health nurses, physicians, and rehabilitation specialists), family members, and non-healthcare caregivers (teachers and trained mediators) can train other school staff members. The most common topics in school training are CPR and first aid (782 instances), followed by airway suctioning with tracheostomy (559). However, only healthcare professionals received training in first aid and CPR. Other topics, like general CWT care, equipment use (inhalers and nebulizers), and oxygen administration, were carried out by experienced family members or caregivers who had prior training.

### School Health Nursing Service-Based Healthcare Model

In this model, school health nurses join the school’s education team, taking over the role of child health coordinator at the school, connecting families, the community, healthcare professionals, and educators. As a member of the school healthcare service, they are responsible for providing direct care to CWT during their time at school. They remain trained, informed, and up-to-date on the most current evidence related to the care of these children. They are supported by a classroom teacher to provide essential communication and information in assessing children’s health and well-being (socialization with other children); they attend educational planning meetings; and they rely on the school principal’s support to review the school health plan recommended for this team^(10,16,17,22,31,33,41)^.

School health nurses maintain a communication channel with children’s primary care physicians, community healthcare services, or other professionals who monitor children’s health, keeping them informed about their condition. This professional is qualified to perform respiratory care including tracheal suctioning, daily peristomal skin assessment, observation for signs of complications (bleeding or infection), and changing the TCT cannula whenever necessary. These care demands are integrated into daily care, adapting them to the school schedule and timetable, for instance, during school arrivals and departures, and during school bus drop-offs and boardings^(16, 17, 18,33,41)^.

Thus, this model combines a set of strategies that facilitate children’s enrollment and retention in school when the time comes for the transition from home to this setting. These strategies include establishing school health nurses’ responsibilities, planning home visits to learn about the daily routine and maintain continuity of care, and encouraging different emergency scenarios with the school team (teachers, administrators, support staff, and firefighters). Furthermore, it informs the school and school transportation staff about children’s needs, such as maintaining humidity in climate-controlled settings (air conditioning or heating), and presents an action plan for activities carried out outside of school (field trips)^(16,17,20,22,26,30,31,41)^.

In relation to direct care, it is a professional with skills and abilities in TCT management and in the daily assessment of children’s condition and stoma, including changing the cannula, reviewing and executing emergency protocols with life-saving measures to ensure a safe and healthy setting in public school settings^(16,17,22,33)^.

In this model, nursing professionals’ relationship with the family consists of creating a parental support group as an opportunity for caregivers to support each other. Nurses learn about children’s condition from parents, assesses the need for training members of the school community in emergency situations, and provides training to family members, teachers, and school staff on procedures such as CPR, TCT care, and pulmonary auscultation. Nurses are responsible for training and delegating tasks, supervising the performance of procedures, and caring for children by school staff. Task delegation builds on the initial and ongoing competence of school staff members to ensure safe and effective care^(16,17,19,22,27,32)^.

These trainings can be conducted by school staff (to whom care is delegated), who are trained by healthcare professionals (school health nurses), who also act as disseminators of knowledge and skills to other members of the school staff. In schools with school health nursing services, the care model is based on school health nurse, and training is essential to ensure safe conditions for children’s continued attendance at school. However, it is necessary that the education professionals who make up the school staff be trained and have access to reliable sources of information on the complex care of this child (Tables 1 and 2). Schools that develop school staff training programs address topics related to suctioning procedures, TCT cannula exchange, CPR, and general child care^(35,40)^.

**Table 2:** Training of education professionals and sources of information on the care of children with tracheostomies at school as a strategy to maintain school attendance^(25, 26, 27, 28, 29, 30)^, 1988–2015.

Variables	N (384)	%
**Trained education professionals**		
School teaching team	150	39
Formal program	76	20
Informal	64	17
With the family	54	14
In a program developed by a university	40	10
**Sources of information on childcare needs and demands**	**N (247)**	**%**
Family	83	34
Family physician	58	23
School health nurse	58	23
Rehabilitation professionals	48	20

Source: Prepared by the authors.

### Family-Based School Healthcare Model

In this family care model, the process of enrolling children with TCT in school begins with clear communication about their special health needs and services to school staff (teachers, administrators, and support staff). The team collaborates in sharing information with children’s future classmates and actively participates in the integration process, sending letters and photos before classes start and in meetings with the principal and teacher. As a stakeholder in children’s safety at school, the team contributes to planning for their retention by proposing training for the school community, offering specific recommendations for accommodations and logistics of school settings, and informing them of health needs that require an emergency plan. Family caregivers express to school administration the need to hire nursing professionals (school health) to train, monitor, and supervise the school staff in health interventions that children require^(9,17,31–32)^.

The family maintains frequent communication with school health nurses when they are present at school. By knowing the child’s complete history and health conditions, they become partners in the decision-making process, involved in developing a documented, written emergency plan to be distributed among those who have contact with the child at school. This plan includes calling the parents in case of an emergency and deciding when it is appropriate to take the child to the hospital and/or community healthcare services. Additionally, they are responsible for providing equipment and supplies necessary for the child’s continued care while at school^(9,17,31–32)^.

In this care model, the parents of a child with TCT act as facilitators in ensuring the child’s right to enroll and remain in school. For instance, family members remain on school grounds and visits the classroom two to three times to monitor children’s condition during their time at school until children are able to self-care for TCT management, airway clearance, cough control, and secretion removal^(18,42)^.

## DISCUSSION

The three school healthcare models discussed in this review highlight school inclusion based on the special health needs and the complex, ongoing, and long-term care required for children with tracheostomy (CWT) outside of the home environment. The findings emphasize the essential role of family members, healthcare providers, and schools in working together in a coordinated manner to ensure that children are enrolled in school and stay there. Each care model developed care strategies in collaboration with the family^(9,18,42)^, in the presence of school health nursing^(17,19,34,36,38)^, and based on the school and healthcare services^(16,22,30,32,39)^. All three school healthcare models present strategies that support the enrollment and retention of CWT in school. Additionally, the models demonstrate the ability to provide targeted care to children at school and to develop and implement an emergency plan under guidelines for respiratory emergencies in CWT. This includes the need for qualified, trained, and skilled personnel to respond immediately, preventing harm to children’s physical well-being. The eligible studies that answered the review question are mostly from international contexts. Anglo-Saxon countries (the United States of America, the United Kingdom, and England) represent most studies, followed by Japan, which is a country with higher levels of economic development that has school healthcare services as an institutional policy. A study from South Africa, a country with lower economic growth, suggests the need to maintain safer schools for CWT.

It is noteworthy that none of the recovered studies originated in Brazil. Since the 1988 Federal Constitution, children’s rights have been enshrined in Article 227, stating that “it is the duty of the family, society, and the State to ensure that children and adolescents, with absolute priority, have the right to life, health, food, education, and leisure, as well as to protect them from all forms of neglect, discrimination, and oppression”^(43)^.

Advances in guaranteeing children’s rights were consolidated with the enactment of Federal Law 8,069 of 1990, the Statute of Children and Adolescents (In Brazilian Portuguese, *Estatuto da Criança e do Adolescente* - ECA)^(44)^, which provides for the comprehensive protection of children and adolescents, who enjoy all the fundamental rights inherent to the human person, without prejudice to comprehensive protection. They are assured of opportunities and facilities that allow for their physical, mental, moral, spiritual, and social development, in conditions of freedom and dignity.

However, the evidence mapping indicates the need to establish a care model that prioritizes school inclusion for CWT. This approach respects their citizenship rights and helps prevent violations that could occur if their mobility needs in urban spaces, such as adapted school transportation, are not addressed, alongside their social assistance needs related to providing essential living conditions, like supplies for ongoing care logistics, and supporting their integration into the community and social life.

In accordance with the Federal Constitution of 1988^(43)^ and the ECA^(44)^, every child is entitled to access education. It is our societal and governmental duty, as members of civil society, to foster critical awareness and to insist that authorities adhere to the legal regulations that safeguard the best interests of children. Furthermore, we must remember that individual actions do not replace the State’s responsibilities, but collective actions mobilize public agents and improve the organization of the process of monitoring the State’s compliance with its constitutional duties, which will lead us to a successful resolution in addressing the problem of school inclusion for children with special healthcare needs.

In Brazil, the Brazilian National Policy for Comprehensive Child Healthcare^(45)^ outlines a set of intra- and intersectoral strategies to include these children in thematic healthcare networks. It focuses on identifying vulnerabilities and risks of harm and illness, acknowledging the specific needs of this population for effective care. There are gaps and few care programs that address the specific needs of children with care demands related to tracheostomy, for example. The policy does not provide care management tailored to children who need to attend school. An inclusive approach is essential, emphasizing strategies and solutions to ensure access to rehabilitation, transportation, and educational services for these children.

Because these children are more vulnerable to complications like cannula obstruction or decannulation, proper care by staff trained in CPR and first aid can prevent such issues^(4,5)^. This helps avoid serious consequences if these events happen at school. The Lucas Law^(46)^ (Law 13,722 of October 4, 2018) mandates basic first aid training for teachers and staff in both public and private elementary and secondary schools, emphasizing the importance of schools being prepared for medical emergencies involving any child. Therefore, this law empowers school staff to handle emergencies effectively when they are adequately trained.

In Brazil, the Brazilian Program of School Health (in Brazilian Portuguese called Programa Saúde na Escola – PSE)^(47)^ is an intersectoral health and education initiative^(46)^ established by Presidential Decree 6,286 of 2007 to support the comprehensive education of students in public basic, primary, and secondary education networks. It is a strategy carried out by the family health team of the Brazilian Health System’s primary care network, conducted through scheduled school visits on a consultative basis. Its actions focus on early detection of high blood pressure, visual and hearing deficits, neglected health issues, and psychosocial disorders.

The PSE is similar to the school-based and healthcare service models, but it faces limitations in meeting the needs of CWT due to their clinical complexity, risk of respiratory emergencies, and the necessity to safeguard children’s physical integrity at school. Both the Federal Constitution and the Law of Guidelines and Bases for National Education^(7)^ guarantee the right to education, meaning the enrollment and retention of children in school. However, achieving school inclusion requires regulation to be enacted through policies, programs, and cultural change within society. Therefore, it is essential to promote reflection on the need for structural and social adjustments to accommodate these CSHCN.

School inclusion prompts us to reflect that, despite all the limitations and barriers that children with TCT may face at school, this will be the place that enables them to be active and free subjects in their own reality. School presents itself as a privileged place for liberation, because only through the production of debates, discussions, and dialogues can we achieve understanding about reality and, thus, promote transformations in society^(48)^.

In Brazil, home-based classes have proven to be an alternative for families who continue the schooling process for CWT at the mandatory school age^(1,7)^. Another aspect is that the PSE focuses on health promotion, disease prevention, and the development of actions that assess vaccination, nutritional, oral, and eye health status, carried out by family health teams according to the school’s demand and specific actions defined by health management in the service area^(47)^. Therefore, the actions they develop do not meet the needs of children living with tracheostomy, creating a challenge for the school, families, and the children themselves.

## CONCLUSION

Strategies to facilitate school enrollment and retention exhibit distinct characteristics across three healthcare models within educational settings: family-based, intersectoral health-education, and outpatient school health. Each model necessitates specific measures to address the care requirements of children with tracheostomy (CWT), including the identification of qualified, trained, and skilled personnel capable of providing safe intervention. It is incumbent upon schools and health institutions, in collaboration with families, to develop appropriate actions and strategies, plan enrollment proactively, and ensure the provision of a safe school environment. This process involves clearly delineating roles and responsibilities, establishing emergency response plans, and implementing suitable protocols to sustain continuous and comprehensive care within the school context.

This review pointed out the lack of studies on care strategies for children with TCT in low-income countries, especially in South America, where collaboration between health and education sectors is still in early development. To improve the quality and relevance of research, it is essential to conduct more studies on the school inclusion of CWT in low- and middle-income countries. Ongoing documentation of actions and strategies for CWT in schools can help showcase success stories that other settings could learn from.

Importantly, the scoping review found few registered practices in low-income countries, which face difficulties in implementing legislation and policies for CSHCN. Potential barriers to including children with TCT in schools include a lack of planning for out-of-hospital follow-up, limited resources to manage their complex health needs, gaps in technical expertise and logistics for school-based care, and poor coordination between schools and healthcare services to support enrollment and retention. The evidence provided offers valuable insights to help include children with TCT in schools, aiding the development of a model suited to the specific context. To protect the right of children with TCT to access school and interact with peers, future research should address gaps in intersectoral health and education strategies focused on the unique ongoing care needs of this group in schools.

## Data Availability

The data for this scoping review are available in the Portuguese version of the doctoral dissertation written by the first author. Minerva UFRJ Database. Access link: https://minerva.ufrj.br/F/VVBF21NFGLQT47PQI1PD9LV29HH6KSR7QXLPLJXXDU27C6ELAH-04989?func=service&doc_library=UFR01&doc_number=000958274&line_number=0001&func_code=WEB-BRIEF&service_type=MEDIA.
