# Supplementary material for: Care strategies for children with tracheostomy at school: a scoping review
Source: Rev Esc Enferm USP. 2025 Nov 3;59:e20250096. doi: 10.1590/1980-220X-REEUSP-2025-0096en (PMC12629661; doi:10.1590/1980-220X-REEUSP-2025-0096en)
Supplement: Translation

# Translation: Estratégias de cuidado para crianças com traqueostomia na escola: revisão de escopo

**DOI:** 10.1590/1980-220X-REEUSP-2025-0096pt

**Authors:** Fernanda Borges Pessanha, Jéssica Renata Bastos Depianti, Thaís Guilherme Pereira Pinheiro Pimentel, Ivone Evangelista Cabral

**Affiliations:** 1 Universidade Federal do Rio de Janeiro, Escola de Enfermagem Anna Nery, Rio de Janeiro, RJ, Brasil.; 2 Universidade do Estado do Rio de Janeiro, Faculdade de Enfermagem, Rio de Janeiro, RJ, Brasil.

**Keywords:** Saúde da Criança, Traqueostomia, Inclusão Escolar, Serviços de Enfermagem Escolar, Serviços de Saúde Escolar

## Abstract

**Objetivo:**

mapear estratégias de cuidados à criança com traqueostomia na escola.

**Método:**

revisão de escopo implementada com a metodologia JBI®. Aplicou-se estratégia de busca abrangente em bases da saúde, educação e literatura cinza no mês de julho de 2024. Os estudos incluídos descreveram diretrizes, políticas de cuidado, experiências dos familiares, profissionais de saúde e de educação sobre cuidados às crianças (6–12 anos) com traqueostomia que frequentam regularmente a escola.

**Resultados:**

os 30 textos recuperados que atenderam aos critérios de elegibilidade apontaram três modelos de atenção em saúde escolar: baseados na família, na intersetorialidade saúde-educação e no ambulatório de saúde escolar.

**Conclusão:**

o êxito da inclusão de crianças com traqueostomia na escola exige articulação intersetorial saúde-educação na formulação de diretrizes. Protocolo de revisão registrado em https://osf.io/3tcg4.

## INTRODUÇÃO

O primeiro consenso da Academia Brasileira de Otorrinolaringologia Pediátrica sobre a abordagem clínica de crianças com traqueostomia (TQT) após a alta hospitalar destaca a inexistência de sistematização de dados sobre essas crianças, a necessidade de insumos para garantir a continuação dos cuidados e o treinamento da equipe de saúde e de leigos para seu acompanhamento. São crianças que mantêm vínculo contínuo com o setor terciário de saúde^(1,2)^. Somado a isso, há pouco reconhecimento social de que elas fazem parte de um grupo que demanda cuidados para sua vida pós-hospitalar^(3)^. Na alta, elas seguem com demandas de cuidados contínuos e complexos, um subgrupo das crianças com necessidades de saúde especiais (CRIANES)^(2,3)^.

A TQT está entre os dispositivos que mais despertam questionamentos em relação à segurança na sua manutenção e integridade da criança^(4,5)^. Além disso, a dependência deste dispositivo pode criar barreiras à vida social dessas CRIANES, pois dependem de cuidados complexos e contínuos para manter a via aérea pérvia. Para tanto, faz-se necessário realizá-los em local com logística favorável às intervenções desses cuidados, como o acesso às tomadas e uma rede elétrica estável^(5)^. No ambiente escolar, aumentam a vulnerabilidade pela dificuldade de transporte escolar seguro, o pessoal capaz de intervir nas emergências respiratórias e as intercorrências associadas à manutenção desses cuidados^(5)^. Ao transitar para a idade escolar, as famílias se preocupam com a inclusão escolar nesse contexto de preparo insuficiente para recebê-las com segurança^(3,6)^.

A Lei de Diretrizes e Bases da Educação Nacional (Lei nº 9.394/1996) garante o atendimento para que o processo de escolarização dessas crianças não seja interrompido, independentemente do contexto, domiciliar ou hospitalar. Quando em casa, a classe domiciliar se destina à criança com impossibilidade de frequentar uma escola regular por problema de locomoção ou internação domiciliar^(7)^.

Nos últimos anos, o problema da inclusão escolar nos Estados Unidos, na Inglaterra e no Japão vem sendo equacionado por meio de estratégias inclusivas que facilitam a continuidade do cuidado^(8–10)^. Nesses países, os direitos à inclusão social, convivência com pares e comunidade escolar são garantidos. Portanto, mobiliza-se a necessidade de um mapeamento abrangente dessas evidências, uma vez que, no Brasil, as políticas e programas vigentes são insuficientes para atender à complexidade de um cuidado contínuo de crianças com TQT na escola^(1)^. O Programa Saúde na Escola (PSE), uma estratégia intersetorial de saúde e educação, possui caráter consultivo, por meio de visitas programadas por profissionais da Estratégia Saúde da Família à escola^(6)^.

As evidências internacionais demonstram que crianças com TQT podem frequentar a escola regular, desde que a escola seja qualificada para prestar os cuidados necessários, incluindo aspiração e desobstrução da cânula, se necessário^(8–10)^. Porém, há uma lacuna de evidências sintetizadas em revisões sistemáticas sobre as estratégias de cuidados facilitadoras da inclusão escolar de CRIANES com TQT; por esta razão, há necessidade de mapeá- las. Em uma busca preliminar no PROSPERO, PubMed, *Cochrane Database of Systematic Reviews* e *JBI Evidence Synthesis*, não se recuperou qualquer tipo de revisão sobre a temática registrada, em andamento ou concluída. Então, definiu-se como objetivo mapear estratégias de cuidado para crianças com TQT no ambiente escolar.

## MÉTODO

### Tipo de Estudo

Esta revisão de escopo foi conduzida no mês de julho de 2024 (última data de aplicação da estratégia de busca em cada base foi no dia 24) seguindo a metodologia JBI^®^ para revisões de escopo^(11)^, e o relatório seguiu as diretrizes de transparência do *Preferred Reporting Items for Systematic Reviews and Meta-Analyses extension for Scoping Reviews* (PRISMA-ScR)^(12)^ (veja Figura 1). Registrou-se o protocolo desta revisão de escopo, *a priori*, na plataforma *Open Science Framework* (https://osf.io/ekp7u/), com o identificador DOI 10.17605/OSF.IO/EKP7U.

**Figura 1:** Fluxograma *Preferred Reporting Items for Systematic Reviews and Meta-Analyses extension for Scoping Reviews* aplicado ao registro da seleção de publicações objeto de análise desta revisão de escopo^(15)^, 2024.

### Pergunta de Revisão

Para a consecução do objetivo da revisão, adotou-se o mnemônico PCC na elaboração da pergunta de pesquisa: P corresponde ao participante/população (profissionais de saúde e educação, gestores escolares e familiares); C corresponde ao conceito (estratégias de cuidados para matrículas e na manutenção de crianças com TQT); e C corresponde ao contexto (escolas regulares). Assim, questiona-se: quais são as estratégias de cuidados adotadas pelos profissionais de saúde e de educação, gestores escolares e familiares para matricular e manter crianças com TQT em escolas regulares?

### Critérios de Elegibilidade

Como critérios de inclusão, consideraram-se artigos cujos participantes eram cuidadores familiares de crianças (de 6 a 12 anos de idade) com TQT que frequentavam a escola, profissionais de saúde em geral, profissionais de enfermagem de saúde escolar, de educação e equipe escolar que atendiam ou cuidavam dessas crianças. Foram considerados estudos que exploraram estratégias de cuidado para garantir a matrícula e a permanência dessas crianças no contexto escolar (sala de aula, pátio, quadra esportiva e transporte escolar). Além disso, incluíram-se também aqueles relacionados à estrutura de serviços de saúde, a exemplo do ambulatório de saúde escolar e serviços de saúde na escola.

Quanto aos tipos de estudos, incluíram-se artigos científicos com abordagens quantitativas, qualitativas e métodos mistos, além de estudos de caso de serviços de saúde escolar para crianças com TQT. Na busca abrangente, incluiu-se ainda a literatura cinza, como teses, dissertações, programas de atenção, diretrizes e editoriais. Nenhum limite de data ou idioma foi aplicado à pesquisa.

### Estratégia de Busca e Seleção dos Estudos

Na metodologia JBI®, a estratégia de busca é implementada em três etapas: 1) procede-se a uma busca limitada às bases de dados PubMed e CINAHL para identificar os termos mais utilizados nos títulos e resumos dos artigos de interesse para a pergunta de revisão; 2) os termos indexados são usados como auxílio na elaboração e no refinamento de uma estratégia de busca sensível e específica; 3) operacionalização da estratégia de busca completa, incluindo os Descritores em Ciências da Saúde, *Medical Subject Headings*, seus respectivos sinônimos, palavras-chave e *entry terms*. Essa estratégia final é adaptada para cada fonte de informação/base de dados. A estratégia de busca aplicada para a biblioteca PubMed é ilustrada na Quadro 1.

**Quadro 1:** Exemplo de uma estratégia de busca aplicada à biblioteca PubMed (MEDLINE).

Fonte de informação	Estratégia de busca em 24 de julho de 2024
PubMed	((Tracheostomy[mh] OR Tracheostom*[tiab]) AND ("Infant"[mh] OR infant*[tiab] OR Child[mh] OR child*[tiab] OR Children[tiab] OR "Disabled Children"[tiab] OR "Children with Disabilities"[tiab] OR "Children with Disability"[tiab] OR "Disabled Child"[tiab] OR "Handicapped Children"[tiab] OR children special health care need*[tiab] OR "exceptional child"[tiab] OR "exceptional children"[tiab] OR "technology dependent child"[tiab] OR "technology dependent children"[tiab] OR "Special Needs Child"[tiab] OR "Special Needs Children"[tiab])) AND ("Mainstreaming, Education"[mh] OR Education Mainstreaming[tiab] OR Educational Mainstreaming[tiab] OR "Return to School"[mh] OR Return School*[tiab] OR "School Health Services"[mh] OR "School Health Services"[tiab] OR "School Based Health Services"[tiab] OR "School Based Services"[tiab] OR "School Health Promotion"[tiab] OR "School Health Service"[tiab] OR "School Based Health Service"[tiab] OR "School Based Health Services"[tiab] OR "School Based Service"[tiab] OR "School Based Services"[tiab] OR Middle School Teacher*[tiab] OR Elementary School Teacher*[tiab] OR "educational institution"[tiab] OR "school building"[tiab] OR "school organisation"[tiab] OR "school organization"[tiab] OR School*[tiab] OR "Elementary Education"[tiab] OR "Elementary School"[tiab] OR "Middle Schools"[tiab] OR "Primary Education"[tiab] OR "Secondary Education"[tiab] OR "intermediate school"[tiab] OR "junior high school"[tiab] OR "junior secondary school"[tiab] OR "lower secondary school"[tiab] OR "Schools"[mh] OR Primary School* OR Secondary School*[tiab] OR "Schools, Nursery"[mh] OR Nursery School*[tiab] OR "Students"[mh] OR Student*[tiab] OR "Education, Special"[mh] OR Special Education*[tiab] OR "Education"[mh] OR Education[tiab] OR Educational Activit*[tiab] OR Literacy Program*[tiab] OR "Teaching"[majr] OR Teaching[tiab] OR Educational[tiab] OR "Schools, Nursing"[mh] OR Nursing School*[tiab] OR Nurse Training School*[tiab] OR school aged[tiab])

As listas de referências de todas as fontes de evidências incluídas foram rastreadas para estudos adicionais. Não houve nenhuma limitação quanto à língua e recorte de tempo. A última busca foi realizada em 24 de julho de 2024. Os bancos de dados pesquisados incluíram PubMed, CINAHL (EBSCOhost), ERIC, PsycInfo, *Web of Science Core Collection*, Scopus/Portal de Periódicos da CAPES, SciELO, LILACS/Biblioteca Virtual em Saúde, *Cochrane Database of Systematic Reviews* e *JBI Evidence Synthesis*. A busca por estudos não publicados e literatura cinzenta incluiu *OpenGrey*, Catálogo de Teses e Dissertações da CAPES, BASE, DART-*Europe*, Plataforma *Dimensions*, e buscas manuais de listas de referências de artigos de texto completo incluídos.

### Extração e Análise de Dados

Para a seleção das fontes de evidência, os registros identificados foram reunidos e carregados no Mendeley™, com as duplicatas sendo removidas. Todo o processo de seleção dos estudos foi realizado com o auxílio da ferramenta Rayyan por dois revisores independentes, evitando o risco de viés na seleção dos estudos de acordo com os critérios de inclusão para a revisão^(13)^. Quaisquer desacordos que surgiram entre os revisores foram resolvidos por meio de discussão ou com um terceiro revisor.

Para a extração dos dados^(14)^, utilizou-se uma ferramenta própria elaborada pelos autores. Dois revisores, de forma independente, extraíram os dados dos resultados dos estudos selecionados e inseriram na ferramenta aqueles que respondiam às estratégias para facilitar a matrícula e manutenção de crianças com TQT em classes escolares regulares. Após esta etapa, os dados foram apresentados ao terceiro revisor, com *expertise* no método e no tema, que selecionou os que melhor atendiam à pergunta desta revisão de escopo para serem submetidos à análise. Nos casos em que houve divergência, os três revisores se reuniram para chegar a um consenso.

As principais descobertas são apresentadas usando análise de conteúdo básica descritiva e organizadas com base nas perguntas de revisão deste estudo. Os dados extraídos são apresentados em formato tabular e diagramático. Um resumo narrativo acompanha os resultados tabulados^(14)^.

## RESULTADOS

Um total de 30 estudos foram retidos para extração de dados e incluídos na revisão. Os resultados da busca e do processo de inclusão do estudo são apresentados de acordo com o fluxograma PRISMA-ScR 2020^(15)^ (Figura 1).

### Caracterização dos Estudos Incluídos

Dos 30 estudos, 24 foram realizados nos Estados Unidos da América^(8,16–38)^, três, na Inglaterra^(9,39,40)^, dois, no Japão^(10,41)^ e um, na África do Sul^(42)^ (Quadro 2). Quanto à natureza dos estudos, sete são qualitativos^(9,10,16–18,41,42)^, sendo seis implementados com entrevistas individuais^(9,10,16–18,42)^ e um grupo focal^(41)^; cinco estudos quantitativos^(34,36–38,40)^ com aplicação de questionários; dois estudos de método misto^(19,35)^; e 16 relatórios de diretrizes de prática clínica^(20–25,32)^, relatos de casos^(26,31)^, modelos consultivos^(8,28,29)^, editorial^(39)^ e revisão não sistematizada de literatura^(33)^. Os estudos quantitativos^(34,36–38,40)^ incluíram uma população de 956 pessoas (enfermeiras de saúde escolar, enfermeiras da atenção primária e hospitalar, professores de escolas primárias, crianças em idade escolar, pais). Nos estudos qualitativos^(9,10,16-18,41,42)^, participaram 839 pessoas (mães, pais, cuidadores, enfermeiras escolares, enfermeiras e professores), e dois relatos de casos^(26,31)^foram implementados com sete crianças. A estratégia de busca recuperou documentos no período de 1988 a 2021, sendo 11 estudos anteriores ao ano 2000^(8,19,21–26,29,30,33,35)^; e após 2000, contabilizaram-se 19 publicações^(9,10,16–18,20,24,27,28,30,31,33,35–39,41,42)^.

**Quadro 2:** Caracterização dos estudos incluídos.

Autor, ano	País	Natureza/tipo de estudo	População/cenário	Objetivo do estudo
Mahomva et al.^(42)^ 2017	África do Sul	Qualitativo	Quatro mães, dois pais; quatro professores/Domicílio e escola	Recomendações para escolas se tornar seguras e acessíveis para crianças com TQT.
Kruger et al.^(16)^ 2009	EUA	Qualitativo	13 enfermeiras/Escola	Desafios e benefícios na interação entre enfermeiros, pais e equipe no cuidado às crianças.
Casey^(17)^ 2002	EUA	Qualitativo	18 enfermeiros escolares/Escola	Papel do enfermeiro escolar que trabalha com alunos clinicamente frágeis em um ambiente escolar.
Mukherjee et al.^(9)^ 2000	Inglaterra	Qualitativo	58 familiares, 34 professores/Escola	Dificuldades encontradas pelos professores no cuidado às crianças.
Rehm and Rohr^(18)^ 2002	EUA	Qualitativo	11 familiares, nove enfermeiros, oito professores, cinco enfermeiros escolares/Domicílio, sala de aula, consultório de enfermagem	Como as famílias, educadores e prestadores de cuidados de saúde garantem a segurança e a saúde de crianças no ambiente escolar.
Shimizu and Katsuda^(10)^ 2015	Japão	Qualitativo	11 professores/Escola	Percepções de professores sobre o papel dos enfermeiros em fornecer cuidados a crianças dependentes de tecnologia nas escolas.
Shimizu and Suzuki^(41)^ 2015	Japão	Qualitativo	21 enfermeiros/Escola, salas de aula, sala de cuidados	Papel dos enfermeiros que cuidam de crianças dependentes de tecnologia nas escolas.
Esperat et al.^(19)^ 1999	EUA	Método misto	618 professores, 43 enfermeiros escolares/Escola	Necessidade dos serviços de educação e saúde nos cuidados de CRIANES nas escolas.
*American Academy Pediatrics: Committee on Injury and Poison Prevention* ^(20)^ 2001	EUA	Relatório de diretrizes de prática	NA	Diretrizes para crianças com TQT durante o transporte.
Graff and Ault^(21)^ 1993	EUA	Diretrizes de prática	NA	Diretrizes para a equipe escolar que trabalha com alunos com necessidades especiais de saúde.
Grundfast et al.^(8)^ 1988	EUA	Programa modelo do centro médico	NA	Integração da criança com TQT no sistema escolar.
Haynie et al.^(22)^ 1989	EUA	Diretrizes de cuidados	NA	Diretrizes informações úteis para transformar os avanços da tecnologia e o compromisso social para as crianças.
Janz et al.^(23)^ 1993	EUA	Diretrizes de cuidados	NA	Discussão das questões da inclusão de CRIANES na sala de aula regular.
Lehr and Greene^(24)^ 2002	EUA	Diretrizes de prática	NA	Questões, problemas e práticas para alunos com necessidades complexas de assistência médica nas escolas.
Lehr^(25)^ 1990	EUA	Diretrizes de prática	NA	Programas educacionais para alunos com necessidades complexas de assistência médica.
Levine^(26)^ 1996	EUA	Estudo de caso	Quatro alunos do sexo masculino e dois do sexo feminino/Escola	Experiência de dependentes de suporte ventilatório que frequentam a escola.
Makrinioti et al.^(39)^ 2021	Inglaterra	Editorial	NA	Abordagem multidisciplinar para enfrentar as dificuldades do retorno à escola para crianças com TQT.
Margolis^(27)^ 2002	EUA	Legislações e políticas	NA	Utilização de leis e políticas dos serviços de saúde escolar.
Palfrey et al.^(28)^ 1992	EUA	Modelo de escola consultiva	NA	Planejamento e procedimentos de treinamento de habilidades.
Parette et al.^(29)^ 1994	EUA	Modelo de inclusão	NA	Papel do enfermeiro pediátrico.
Porter et al.^(30)^ 2013	EUA	Preparação para emergências	NA	Instruções específicas de atendimento de emergência para alunos na escola.
Raymond^(31)^ 2009	EUA	Estudo de caso	Menino de 6 anos/Escola	Processo de integração, papéis da equipe e implicações para a prática de enfermagem escolar.
Smith and Leatherby^(32)^ 1992	EUA	Diretrizes	NA	Prestação de serviços para alunos para procedimentos especiais de cuidados de saúde na escola.
Toothaker and Cook^(33)^ 2018	EUA	Revisão não sistematizada	NA	Habilidades de enfermeiro escolar para cuidar de alunos com TQT.
Mulligan-Ault et al.^(34)^ 1988	EUA	Quantitativo	150 professores/Escola	O papel do enfermeiro escolar.
Patel et al.^(35)^ 2009	EUA	Método misto	23 crianças/Hospital	Experiência escolar de crianças com TQT.
Pufpaff et al.^(36)^ 2015	EUA	Quantitativo	76 enfermeiros escolares/Escola	Colaboração entre enfermeiro da escola e professor.
Smith et al.^(40)^ 2003	Reino Unido	Quantitativo	11 familiares e o cuidador da criança na escola/Hospital	Identificação das crianças que frequentam escolas e o apoio recebido.
Heller et al.^(37)^ 2000	EUA	Quantitativo	342 professores, 49 profissionais de saúde não enfermeiros, 21 enfermeiros, dez administradores escolares/Escola	Treinamento ao pessoal não médico em habilidades de saúde para um ambiente seguro.
Sapien and Allen^(38)^ 2001	EUA	Quantitativo	230 enfermeiros escolares/Escola	Preparação das escolas públicas para necessidades emergentes de saúde em conformidade com as diretrizes.

Legenda: EUA – Estados Unidos da América. NA – não se aplica; TQT – traqueostomia; CRIANES – crianças com necessidades de saúde especiais. Fonte: elaborada pelas autoras.

As estratégias adotadas pelos profissionais de saúde, de educação e cuidadores familiares para matricular e manter os cuidados das crianças com TQT em escolas regulares se organizam em três modelos, como demonstrado na Figura 2.

**Figura 2:** Modelos de atenção à saúde escolar de crianças com necessidades de especiais de saúde vivendo com traqueostomia.

Fonte: Elaborada pelas autoras.

### Modelo de Atenção à Saúde escolar Baseado na Parceria entre Escola e Serviço de Saúde

O modelo de atenção à saúde escolar baseado na escola e no serviço de saúde foca na parceria entre o serviço de referência para a continuidade dos cuidados à criança, a escola e os recursos da comunidade. Juntos, planejam estratégias e ações para assegurar a matrícula e sua permanência no ambiente escolar. Para tanto, destacam-se: a implementação de recomendações de profissionais de saúde para atender às demandas específicas da criança na escola e providenciar os equipamentos necessários; a identificação de situações de emergências relacionadas à TQT e formas de contato com a equipe de emergência (ambulância ou resgate ou pronto-socorro); a contratação de pessoal qualificado para o cuidado; a organização de uma visita do serviço de saúde à escola; e a previsão de sessão de treinamento da equipe escolar pelo serviço de saúde^(8,9,18–19,21–24,26,28,29,32,42)^.

Para isso, antes do início do ano escolar, preveem-se o envolvimento dos profissionais de saúde na gestão de emergências na escola e a disponibilização de informações sobre a criança para a comunidade escolar. Prioriza-se o desenvolvimento de políticas, protocolos e algoritmos, com as informações essenciais sobre as condições de saúde da criança a serem antecipadas e comunicadas à comunidade escolar. Agenda-se uma reunião individual com a criança, pais e responsáveis na escola^(19–20,22,24–26,30,32,42)^.

O serviço de saúde compartilha com a escola as especificidades do cuidado à criança com a TQT, o que pode se constituir em emergência, e quais procedimentos devem ser realizados em uma intervenção de emergência. O atendimento é devidamente documentado caso haja necessidade de acionar os serviços de saúde da comunidade*.* Diante dessas informações, as escolas adaptam suas logísticas para atender às demandas de saúde especiais da criança, provendo espaço fora da sala de aula quando não se sentirem bem, tais como sala privativa, para a realização de procedimentos, climatizada, com controle de temperatura, acesso à água para limpeza de material e lavagem das mãos, local de armazenamento de equipamentos e suprimentos, tomadas elétricas, e fontes alternativas de energia^(8,9,21–24,28,30,31)^.

Logo que o planejamento de transição para matrícula da criança foi concluído e as medidas adaptativas ambientais foram implementadas, iniciaram-se as estratégias para sua permanência na escola, com avaliação após um mês de inclusão escolar*.* Alguns estudos recomendam, como parte da política de inclusão escolar de criança com TQT, que a inserção segura requer um profissional de enfermagem disponível na escola, sempre que a criança precisar. Para garantir a saúde e a sua segurança, há médicos que defendem a acomodação da criança próxima à mesa do professor, mantendo um olhar atento sobre seu comportamento. É preciso estar alerta para informar aos pais sobre riscos potenciais de infecção ou sintomas precoces, para que possam manter as crianças em casa ou levá-las ao atendimento de saúde logo no início de uma infecção^(9,16–18,21,42)^.

As escolas contam com a colaboração do profissional de saúde especializado para prescrever e indicar diretrizes padronizadas de cuidados a essa criança. Além disso, identificam um membro da equipe de saúde como pessoa de ligação que, em conjunto com os pais da criança, informa sobre suas condições de saúde e toma decisões sobre o plano de cuidado. Indicam quem é responsável pela compra e manutenção dos equipamentos, descarte de resíduos de saúde e fornecimento de suprimentos necessários aos cuidados de saúde da criança na escola^(8,9,20–22,26,40)^.

Para a permanência da criança na escola, a equipe da comunidade escolar recebe um treinamento do serviço de saúde que pode ser realizado na escola ou na unidade de saúde da comunidade. O treinamento é realizado pelos profissionais da internação domiciliar, profissionais com experiência clínica em cuidados pediátricos, pessoas com capacitação e conhecimento sobre os direitos da criança (legislações), educadores previamente treinados, e familiares cuidadores com experiência em procedimentos específicos realizados em seus próprios filhos^(8,18,21–31,39,40)^. Nesse sentido, o treinamento é ministrado por profissionais certificados para capacitar professores, gestores, pessoal assistente, funcionários do transporte escolar, coordenador, profissional de enfermagem em saúde escolar (recém-contratado) e colegas de classe; ou seja, todas as pessoas que mantêm contato regular com a criança. Quando a equipe da escola é treinada, os procedimentos específicos no manejo da TQT no ambiente escolar são realizados de forma segura e apropriada.

O treinamento é baseado em competências e habilidades no gerenciamento de problemas e formalizado segundo as necessidades dos membros da comunidade escolar e daqueles cuidadores que prestam os cuidados diretos à criança na escola. São utilizados recursos gráficos (vídeos), sessões de ensino, perguntas e respostas, informações verbais adicionais das famílias, solução de problemas, e visitas regulares com sessões de atualização para oferecer suporte e manter suas habilidades. A escola e o serviço de saúde documentam e reforçam o treinamento de tempos em tempos, treinando pelo menos duas ou três pessoas para que haja apoio disponível^(4,8,10,11,13,14,18,20,25,27,29,31,39,40)^.

Em síntese, as evidências qualitativas apontam como temas abordados nos treinamentos, regulamentações sobre quem pode realizar procedimentos especializados e complexos na sala de aula, acesso a recursos comunitários e formas de contato em casos de urgências. Incluíram-se os conhecimentos básicos sobre a TQT aplicados ao contexto escolar, plano de emergência, sinais de desconforto, condição respiratória e geral da criança para atender às necessidades de saúde especiais.

Evidências quantitativas indicadas na Tabela 1 destacam os temas reanimação cardiopulmonar (RCP), primeiros-socorros e cuidados gerais com a TQT. A necessidade de treinamento inclui procedimentos de aspiração da via aérea pela TQT, uso de dispositivos e administração de oxigênio^(8,18,21–25,27–29,31,32,42)^.

**Tabela 1:** Temas abordados nos treinamentos da equipe da escola, sob responsabilidade do serviço de saúde, de enfermagem em saúde escolar e da própria escola, como estratégia para a manutenção de crianças com traqueostomia na escola^(34,35,37,38,40)^,1988–2015.

Temas abordados no treinamento	Profissional de saúde^(37,38)^ (n = 572)			Familiar e cuidador não profissional (n = 643)		
	Profissional de enfermagem em saúde escolar (n = 1.078)	Médico (n = 88)	Profissionais de reabilitação (n = 132)	Serviço de saúde (n = 159)	Familiar (n = 444)	Cuidador (professor + mediador) (n = 199)
Primeiros-socorros^(37)^		256		–	–	–
Reanimação cardiopulmonar^(37,38)^		526		–	–	–
Aspiração^(37)^ n = 559	283	24	38	48	113	53
Cuidados gerais com a traqueostomia^(37)^ n = 539	272	17	30	49	128	43
Uso de inalador/nebulizador^(37)^ n = 516	250	23	23	28	127	65
Administração de oxigênio^(37)^ n = 486	273	24	41	34	76	38

Fonte: Elaborada pelas autoras.

Na Tabela 1, os profissionais de saúde (enfermagem de saúde escolar, médicos e profissionais de reabilitação), familiares e cuidadores não profissionais de saúde (professor e mediadores treinados), que estão capacitados, podem treinar os demais membros da equipe escolar. Os temas mais recorrentes no treinamento na escola são RCP e primeiros-socorros (782 ocorrências), seguidos pela aspiração de vias aéreas pela TQT (559). Porém, somente os profissionais de saúde realizaram treinamentos sobre primeiros-socorros e RCP. Os demais temas, como cuidados gerais com a TQT, uso de equipamentos (inalador e nebulizador) e administração de oxigênio, foram implementados por familiares com experiência ou cuidadores previamente capacitados.

### Modelo de Atenção à Saúde Baseado no Serviço de Enfermagem de Saúde Escolar

Nesse modelo, o profissional de enfermagem em saúde escolar integra a equipe de educação da escola, assumindo a função de coordenador de saúde da criança na escola, conectando a família, a comunidade, os profissionais de saúde e os educadores. Como membro do serviço de saúde escolar, é responsável pela prestação de cuidados diretos à criança com TQT durante o tempo de permanência dela na escola. Mantém-se capacitado (treinamentos), informado e atualizado sobre as evidências mais atuais relacionadas ao cuidado a essas crianças. Tem o apoio do professor de sala de aula para oferecer comunicação e informações essenciais na avaliação da saúde e bem-estar da criança (socialização com outras crianças); frequenta as reuniões de planejamento educacional; e conta com o apoio do diretor da escola para revisar o planejamento de saúde na escola que é recomendado para essa equipe^(10,16,17,22,31,33,41)^.

O profissional de enfermagem em saúde escolar mantém um canal de comunicação com os médicos de referência de criança, com serviços de saúde da comunidade ou com outros profissionais que acompanham a saúde da criança, informando-se sobre as condições da criança. É um profissional qualificado para realizar os cuidados respiratórios na aspiração traqueal, avaliação diária da pele periestoma, observação de sinais de complicações (sangramento ou infecção) e troca da cânula de TQT sempre que necessário. Essas demandas de cuidados integram os cuidados diários, ajustando-os ao cronograma e horário escolar, por exemplo, na entrada e saída da escola, no desembarque e embarque do ônibus escolar^(16-18,33,41)^.

Dessa maneira, esse modelo reúne um conjunto de estratégias que facilitam a matrícula da criança e sua permanência na escola, quando chega o momento da transição da casa para esse ambiente. Entre essas estratégias, destacam-se o estabelecimento das atribuições do profissional de enfermagem em saúde escolar, o planejamento de visitas domiciliares para conhecimento da rotina diária e manutenção da continuidade do cuidado, e a simulação de diferentes cenários de emergências com a equipe escolar (professores, gestores, pessoal de apoio, brigadista). Além disso, informa a equipe da escola e do serviço de transporte escolar sobre as necessidades da criança, como é o caso da manutenção da umidade de ambientes climatizados (ar-condicionado ou calefação), e apresenta um plano de ação para as atividades realizadas fora da escola (excursão)^(16,17,20,22,26,30,31,41)^.

Em relação aos cuidados diretos, é um profissional com competências e habilidades no manejo da TQT e na avaliação diária da condição da criança e do estoma, incluindo a troca da cânula, revisando e executando protocolos de emergência com medidas de salvamento para garantir um ambiente seguro e saudável no ambiente escolar público^(16,17,22,33)^.

Nesse modelo, a relação do profissional de enfermagem com a família consiste na criação de grupo de apoio parental como uma oportunidade para os cuidadores se apoiarem mutuamente. Ele se informa sobre as condições da criança com os pais, verifica a necessidade de treinamento dos membros da comunidade escolar sobre situações emergenciais, realizando treinamento com familiares, professores e equipe escolar na realização de procedimentos como RCP, cuidados com a TQT e ausculta pulmonar. Responsabiliza-se pelo treinamento e delegação de tarefas, supervisão do desempenho na realização de procedimentos, e cuidados dessa criança pelo pessoal da comunidade escolar. A delegação de tarefas se baseia na competência inicial e continuada de membros da equipe escolar para garantir cuidados seguros e eficazes^(16,17,19,22,27,32)^.

Esses treinamentos podem ser conduzidos por pessoal da própria escola (a quem será delegado a tarefa do cuidado), que é treinado por profissionais de saúde (profissional de enfermagem de saúde escolar), que também atua como disseminador de conhecimentos e habilidades para outros membros da equipe escolar. Naquelas escolas que há serviços de enfermagem em saúde escolar, o modelo de atenção é baseado no profissional de enfermagem de saúde escolar, e o treinamento é essencial para que se garantam condições seguras à permanência dessa criança na escola. Porém, é necessário que os profissionais de educação que compõem a equipe escolar sejam treinados, bem como ter acesso a fontes de informações confiáveis sobre os cuidados complexos dessa criança (Tabelas 1 e 2). As escolas que desenvolvem programas de treinamento da equipe escolar abordam temas relacionados a procedimentos de aspiração, troca de cânula da TQT, RCP e cuidados gerais da criança^(35,40)^.

**Tabela 2:** Treinamento de profissionais de educação e fontes de informações sobre cuidados de crianças com traqueostomia na escola como estratégia para manter a frequência escolar^(25–30)^, 1988–2015.

Variáveis	N (384)	%
**Profissionais de educação treinados**		
Equipe de professores da escola	150	39
Programa formal	76	20
Informal	64	17
Com a família	54	14
Em programa desenvolvido por universidade	40	10
**Fontes de informações sobre as necessidade e demandas de cuidados à criança**	**N (247)**	**%**
Família	83	34
Médico de família	58	23
Profissional de enfermagem de saúde escolar	58	23
Profissionais de reabilitação	48	20

Fonte: elaborada pelas autoras.

### Modelo de Atenção à Saúde Escolar Baseado na Família

Nesse modelo de atenção à família, inicia-se o processo de matrícula da criança com TQT na escola, comunicando claramente as informações sobre suas necessidades de saúde especiais e de serviços à equipe escolar (professores, gestores e pessoal de apoio). A equipe atua de forma colaborativa no compartilhamento de informações aos futuros colegas de classe da criança e participa ativamente do processo de integração, enviando cartas e fotos antes do início das aulas e nas reuniões com o diretor e professor. Como parte interessada pela segurança da criança na escola, contribui com o planejamento para sua permanência, ao propor um treinamento para a comunidade escolar, oferecendo recomendações específicas para as acomodações e logísticas do ambiente escolar, e informando sobre as necessidades de saúde que requerem um plano de emergência. Os familiares cuidadores expressam à direção da escola a necessidade de contratação de profissionais de enfermagem (de saúde escolar) para treinar, monitorar e supervisionar a equipe escolar nas intervenções de saúde que a criança precisa^(9,17,31,32)^.

A família mantém uma comunicação frequente com o profissional de enfermagem de saúde escolar, quando este está presente na escola. Por conhecer todo o histórico e as condições de saúde da criança, torna-se parceira no processo de tomada de decisão, envolvendo-se na elaboração de um plano de emergência, documentado e por escrito, a ser distribuído entre aqueles que mantêm contato com essa criança na escola. Esse plano inclui telefonar para os pais em caso de emergência e decidir quando é apropriado levar a criança ao hospital e/ou para serviços de saúde da comunidade. Além disso, são responsáveis por fornecer equipamentos e suprimentos necessários à continuidade do cuidado à criança enquanto estiver na escola^(9,17,31,32)^.

Nesse modelo de atenção, os pais da criança com TQT atuam como facilitadores na garantia do direito à matrícula e permanência dessa criança na escola. Por exemplo, o familiar permanece no espaço escolar e visita a sala de aula de duas a três vezes para acompanhar a condição da criança durante sua permanência na escola até que ela seja capaz de se autocuidar no manejo da TQT, na limpeza das vias aéreas, no controle da tosse e na remoção de secreções^(18,42)^.

## DISCUSSÃO

Os três modelos de atenção à saúde escolar mapeados nesta revisão apontam para uma inclusão escolar fundamentada nas necessidades de saúde especiais e na natureza complexa dos cuidados contínuos e prolongados de crianças com TQT para além do ambiente da casa. Os resultados indicam o papel fundamental dos familiares, dos serviços de saúde e da escola, atuando de forma colaborativa e organizada para garantir a matrícula e sua permanência na escola.

Cada modelo de atenção desenvolveu estratégias de cuidados pensadas em conjunto com a família^(9,18,42)^, com a presença da enfermagem de saúde escolar^(17,19,34,36,38)^ e baseado na escola e no serviço de saúde^(16,22,30,32,39)^. Todos os três modelos de atenção à saúde escolar apresentaram estratégias facilitadoras para a matrícula e manutenção da criança com TQT na escola. Além disso, os modelos demonstram a capacidade de fornecer cuidados específicos à criança na escola e estabelecer e executar plano de emergência em conformidade com as orientações de emergências respiratórias à criança com TQT. Prevê a necessidade de dispor de pessoal qualificado, treinado e capacitado para atuar imediatamente, evitando danos à integridade física da criança.

Os estudos elegíveis que responderam à pergunta de revisão são, em sua maioria, de procedência de contextos internacionais. Os países anglo-saxões (Estados Unidos da América, Reino Unido e Inglaterra) representam a maioria dos estudos, seguidos pelo Japão, ou seja, países com maior nível de desenvolvimento econômico que possuem serviços de saúde escolar como política institucional. No estudo da África do Sul, país com menor nível de desenvolvimento econômico, as evidências apontam para a necessidade de manter escolas mais seguras para crianças com TQT.

Destaca-se o fato de não haver entre os estudos recuperados nenhum de origem no Brasil. Desde a Constituição Federal de 1988, a criança tem seus direitos previstos no Art. 227, sendo “dever da família, sociedade e Estado assegurar à criança e ao adolescente, com absoluta prioridade, o direito à vida, à saúde, à alimentação, à educação, ao lazer, além de colocá-los a salvo de toda forma de negligência, discriminação e opressão”^(43)^.

Os avanços na garantia de direitos à criança se consolidam com a promulgação da Lei Federal nº 8.069, 1990, do Estatuto da Criança e do Adolescente (ECA)^(44)^, que dispõe sobre a proteção integral à criança e ao adolescente, que usufruem de todos os direitos fundamentais inerentes à pessoa humana, sem prejuízo da proteção integral. A elas são asseguradas oportunidades e facilidades, que lhes facultam o desenvolvimento físico, mental, moral, espiritual e social, em condições de liberdade e de dignidade.

Porém, o mapeamento das evidências aponta para a necessidade de implementação de modelo de atenção que favoreça a inclusão escolar de criança com TQT. Desse modo, respeitam-se seus direitos de cidadania, evitando-se que sejam violados caso não sejam atendidas as necessidades de locomoção no espaço urbano (transporte escolar adaptado), as necessidades de assistência social no provimento de condições materiais de existência (insumos para a logística do cuidado contínuo) e na facilitação de sua inclusão na comunidade e convívio social.

Segundo a Constituição Federal de 1988^(43)^ e o ECA^(44)^, toda criança tem o direito de frequentar a escola. É de nossa responsabilidade social e política, enquanto sociedade civil, desenvolver consciência crítica e exigir das autoridades o cumprimento das diretrizes legais que protegem o melhor interesse da criança. Além disso, devemos lembrar que ações individuais não substituem as tarefas do Estado, mas as ações coletivas mobilizam agentes públicos e organizam melhor o processo de fiscalização do cumprimento ou não do Estado, dos seus deveres constitucionais, que nos levará a bom termo no enfrentamento do problema da inclusão escolar da criança com demandas de cuidados de saúde especiais.

No Brasil, a Política Nacional de Atenção Integral à Saúde da Criança^(45)^ articula um conjunto de estratégias intra e intersetoriais para inclusão dessas crianças nas redes temáticas de atenção à saúde, mediante a identificação de vulnerabilidades e risco de agravos e adoecimento, reconhecendo as especificidades deste público para uma atenção resolutiva. Existem lacunas e poucos programas de atenção voltados para a especificidade da criança com demandas de cuidados associados à TQT, por exemplo. A política não garante uma gestão de cuidado voltada para as necessidades dessas crianças que precisam ir à escola. É necessário um olhar inclusivo que aponte estratégias e resoluções para garantir o acesso a serviços de reabilitação, transporte e educação para esse grupo infantil.

Especialmente, por tratar-se de crianças com maior risco de complicações, tais como obstrução da cânula ou decanulação, as mesmas podem ser evitadas com o cuidado adequado prestado por equipe treinada em RCP e primeiros-socorros^(4,5)^. Assim, evitam-se consequências catastróficas, quando esses eventos ocorrerem na escola. A Lei Lucas^(46)^ (Lei nº 13.722, de 4 de outubro de 2018) torna obrigatória a capacitação em noções básicas de primeiros-socorros de professores e funcionários de estabelecimentos de ensino públicos e privados de educação básica, apontando a necessidade que o ambiente educacional vem enfrentando para lidar com intercorrências médicas a qualquer criança. Nesse sentido, esta lei respalda a equipe da escola para atuar em emergências, desde que devidamente capacitada.

No Brasil, o PSE^(47)^ é uma iniciativa intersetorial saúde e educação^(46)^ instituída pelo Decreto Presidencial nº 6.286 de 2007 para contribuir com a formação integral de estudantes das redes públicas da educação básica, fundamental e média. É uma estratégia implementada pela equipe de saúde da família da rede de atenção básica do Sistema Único de Saúde, executada em caráter consultivo por meio de visitas agendadas na escola. Suas ações visam à detecção precoce de hipertensão arterial, déficits visuais e auditivos, problemas de saúde negligenciados e distúrbios psicossociais.

O PSE se assemelha ao modelo baseado na escola e no serviço de saúde, mas com limitações para suprir as necessidades da criança com TQT, por sua complexidade clínica, risco de emergência respiratória e proteção à integridade física da criança na escola.

Tanto a Constituição Federal quanto a Lei de Diretrizes e Bases da Educação Nacional^(7)^ preveem o direito à educação, ou seja, matrícula e permanência da criança na escola. Contudo, a inclusão escolar implica a necessidade de a regulamentação ser efetivada em políticas, programas e pela mudança de cultura de uma sociedade. Para isso, faz-se necessário promover reflexões acerca das necessidades de adequações estruturais e sociais para o acolhimento dessas CRIANES.

A inclusão escolar nos faz refletir que apesar de todas as limitações e barreiras que as crianças com TQT poderão enfrentar na escola, esse será o lugar que as tornarão sujeito ativo e livre em sua própria realidade. A escola se apresenta como local privilegiado à libertação, pois somente através da produção de debates, discussões e diálogos é que se pode alcançar a compreensão acerca da realidade e, assim, promover transformações na sociedade^(48)^.

No Brasil, as classes domiciliares têm se mostrado uma alternativa para famílias que mantêm o processo de escolarização de crianças com TQT na idade obrigatória de frequentar a escola^(1,7)^. Outro aspecto é o fato de o PSE focar na promoção da saúde, prevenção de doenças e desenvolvimento de ações que avaliam a situação vacinal, nutricional, de saúde bucal e ocular, realizadas por equipes de saúde da família, conforme a demanda da escola e ações específicas definidas pela gestão de saúde na área de atendimento^(47)^. Portanto, as ações que desenvolvem não cobrem as necessidades de crianças que vivem com TQT, representando um desafio para a escola, as famílias e as próprias crianças.

## CONCLUSÃO

As estratégias facilitadoras da matrícula e da permanência na escola revelaram características distintas mapeadas em três modelos de atenção à saúde escolar: baseado na família, na intersetorialidade saúde-educação e no ambulatório de saúde escolar. Cada um deles apresenta especificidades para atender às demandas de cuidado às crianças com TQT, identificando pessoal habilitado, treinado e capacitado para intervir com segurança. Cabe às instituições escolar e de saúde, em parceria com a família, estabelecer ações e estratégias, planejar antecipadamente a matrícula, e garantir condições de permanência segura na escola, com clara definição de papéis e responsabilidades, além de definir planos de emergência e protocolos apropriados à manutenção dos cuidados contínuos e complexos na escola.

Esta revisão apontou a escassez de estudos sobre as estratégias de cuidados para crianças com TQT em países de baixa renda, especialmente na América do Sul, onde a colaboração entre os setores de saúde e educação ainda é emergente. Para aumentar a qualidade e a relevância dos resultados de pesquisa, é fundamental realizar mais investigações sobre a inclusão escolar de crianças com TQT em países de baixa e média renda. A documentação contínua das ações e estratégias direcionadas a crianças com TQT nas escolas pode contribuir para evidenciar casos de sucesso dos quais outros contextos podem se beneficiar.

É importante ressaltar que a revisão de escopo identificou escassas práticas registradas em países de baixa renda, que enfrentam desafios na implementação das legislações e políticas voltadas para CRIANES. As barreiras potenciais à inclusão de crianças com TQT nas escolas incluem a falta de planejamento para ações de acompanhamento fora do hospital, recursos limitados para lidar com a complexidade da saúde dessas crianças, lacunas na competência técnica e na logística para atendê-las na escola, além da falta de coordenação entre a escola e os serviços de saúde para garantir a matrícula e a permanência na instituição. As evidências apresentadas oferecem informações valiosas para facilitar a inclusão de crianças com TQT na escola, contribuindo para a construção de um modelo alinhado ao contexto. Para assegurar o direito de acesso de crianças com TQT à inclusão escolar e à convivência com seus pares, é necessário que futuras pesquisas sejam realizadas para preencher as lacunas sobre estratégias intersetoriais saúde e educação dirigidas às especificidades do cuidado contínuo desse grupo infanto-juvenil nas escolas.

## Data Availability

Os dados da presente revisão de escopo estão disponíveis na versão em português da tese de doutorado de autoria da primeira autora. Base Minerva UFRJ. Link de acesso: https://minerva.ufrj.br/F/VVBF21NFGLQT47PQI1PD9LV29HH6KSR7QXLPLJXXDU27C6ELAH-04989?func=service&doc_library=UFR01&doc_number=000958274&line_number=0001&func_code=WEB-BRIEF&service_type=MEDIA.

# Translation: Estrategias de cuidado de niños con traqueotomía en la escuela: una revisión de alcance

**DOI:** 10.1590/1980-220X-REEUSP-2025-0096es

**Authors:** Fernanda Borges Pessanha, Jéssica Renata Bastos Depianti, Thaís Guilherme Pereira Pinheiro Pimentel, Ivone Evangelista Cabral

**Affiliations:** 1 Universidade Federal do Rio de Janeiro, Escola de Enfermagem Anna Nery, Rio de Janeiro, RJ, Brasil.; 2 Universidade do Estado do Rio de Janeiro, Faculdade de Enfermagem, Rio de Janeiro, RJ, Brasil.

**Keywords:** Salud Infantil, Traqueotomía, Integración Escolar, Servicios de Enfermería Escolar, Servicios de Salud Escolar

## Abstract

**Objetivo:**

Mapear las estrategias de cuidado de niños con traqueostomía en la escuela.

**Método:**

Revisión de alcanze implementada mediante la metodología JBI®. Se aplicó una estrategia de búsqueda exhaustiva en bases de datos de salud, educación y literatura gris en julio de 2024. Los estudios incluidos describieron guías, políticas de cuidado y las experiencias de familiares, profesionales de la salud y de la educación en relación con la salud de niños (de 6 a 12 años) con traqueostomía que asisten regularmente a la escuela.

**Resultados:**

Los 30 textos recuperados que cumplieron con los criterios de elegibilidad identificaron tres modelos de atención de salud escolar: familiar, intersectorial de educación para la salud y ambulatorio de salud escolar.

**Conclusión:**

La inclusión exitosa de niños con traqueostomía en las escuelas requiere la coordinación intersectorial de educación para la salud en la formulación de directrices. El protocolo de revisión está registrado en https://osf.io/3tcg4

## INTRODUCCIÓN

El primer consenso de la Academia Brasileña de Otorrinolaringología Pediátrica sobre el enfoque clínico de los niños con traqueotomía (TQT) después del alta hospitalaria destaca la falta de sistematización de los datos sobre estos niños, la necesidad de insumos para garantizar la continuidad de los cuidados y la formación del personal sanitario y de los legos para su seguimiento.

Se trata de niños que mantienen un vínculo continuo con el sector terciario de la salud^(1,2)^. A esto se suma el escaso reconocimiento social de que forman parte de un grupo que requiere cuidados para su vida posthospitalaria^(3)^. Al ser dados de alta, siguen necesitando cuidados continuos y complejos, lo que los convierte en un subgrupo de niños con necesidades de atención y cuidados de salud especiales (NANEAS, para Latin America)^(2,3)^.

La TQT es uno de los dispositivos que más dudas suscita en cuanto a la seguridad de su mantenimiento y la integridad del niño^(4,5)^. Además, la dependencia de este dispositivo puede crear barreras a la vida social de estos NANEAS, ya que dependen de cuidados complejos y continuos para mantener las vías respiratorias permeables. Por ello, es necesario realizarlos en un lugar con una logística favorable para estas intervenciones, como el acceso a enchufes y una red eléctrica estable^(5)^. En el entorno escolar, la vulnerabilidad aumenta debido a la dificultad de transporte escolar seguro, la falta de personal capaz de intervenir en emergencias respiratorias y las complicaciones asociadas al mantenimiento de estos cuidados^(5)^. Al llegar a la edad escolar, las familias se preocupan por la inclusión escolar en este contexto de preparación insuficiente para recibir a los niños con seguridad^(3,6)^.

La ley brasileña de Directrices y Bases de la Educación Nacional (Ley n.º 9.394/1996) garantiza la atención para que el proceso de escolarización de estos niños no se interrumpa, independientemente del contexto, ya sea domiciliario u hospitalario. Cuando están en casa, la clase domiciliaria está destinada a los niños que no pueden asistir a una escuela regular por problemas de movilidad o hospitalización domiciliaria^(7)^.

En los últimos años, el problema de la inclusión escolar en Estados Unidos, Inglaterra y Japón se ha abordado mediante estrategias inclusivas que facilitan la continuidad del cuidado^(8–10)^. En estos países, se garantizan los derechos a la inclusión social, la convivencia con los compañeros y la comunidad escolar. Por lo tanto, es necesario realizar un mapeo exhaustivo de estas evidencias, ya que, en México, las políticas y programas vigentes son insuficientes para atender la complejidad del cuidado continua de los niños con TQT en la escuela^(1)^. El Programa Salud en la Escuela (PSE), una estrategia intersectorial de salud y educación, tiene carácter consultivo, a través de visitas programadas a la escuela por profesionales de la Estrategia de Salud Familiar^(6)^.

Las evidencias internacionales demuestran que los niños con TQT pueden asistir a la escuela regular, siempre que la escuela esté capacitada para proporcionar los cuidados necesarios, incluyendo la aspiración y la desobstrucción de la cánula, si es necesario^(8–10)^. Sin embargo, existe una falta de evidencia sintetizada en revisiones sistemáticas sobre las estrategias de atención que facilitan la inclusión escolar de NANEAS con TQT; por esta razón, es necesario mapearlas. En una búsqueda preliminar en PROSPERO, PubMed, *Cochrane Database of Systematic Reviews* y *JBI Evidence Synthesis*, no se recuperó ningún tipo de revisión sobre el tema registrado, en curso o concluida. Por lo tanto, se definió como objetivo mapear estrategias de atención para niños con TQT en el entorno escolar.

## MÉTODO

### Tipo De Estudio

Esta revisión de alcance fue realizada en el mes de julio de 2024 (la última actualización de la estrategia de búsqueda en cada base de datos ocurrió el día 24), siguiendo la metodología JBI® para este tipo de revisión^(11)^, y el informe siguió las directrices de transparencia de los *Preferred Reporting Items for Systematic Reviews and Meta-Analyses extension for Scoping Reviews* (PRISMA-ScR)^(12)^ (Figura 1). El protocolo de esta revisión del alcance se registró, *a priori*, en la plataforma *Open Science Framework* (https://osf.io/ekp7u/), con el identificador DOI 10.17605/OSF.IO/EKP7U.

**Figura 1:** Flujograma *Preferred Reporting Items for Systematic Reviews and Meta-Analyses extension for Scoping Reviews* aplicado al registro de la selección de publicaciones objeto de análisis de esta revisión de alcance^(15)^, 2024.

### Pregunta de Revisión

Para alcanzar el objetivo de la revisión, se adoptó el mnemónico PCC en la elaboración de la pregunta de investigación: P corresponde al participante/población (profesionales de la salud y la educación, directores escolares y familiares); C corresponde al concepto (estrategias de atención para la matriculación y la permanencia de niños con TQT); y C corresponde al contexto (escuelas regulares). Así, se plantea la siguiente pregunta: ¿cuáles son las estrategias de atención adoptadas por los profesionales de la salud y la educación, los directores de escuelas y los familiares para matricular y mantener a los niños con TQT en escuelas regulares?

### Criterios de Elegibilidad

Como criterios de inclusión, se consideraron artículos cuyos participantes eran cuidadores familiares de niños (de 6 a 12 años de edad) con TQT que asistían a la escuela, profesionales de la salud en general, profesionales de enfermería escolar, de educación y personal escolar que atendían o cuidaban a estos niños. Se consideraron estudios que exploraban estrategias de cuidado para garantizar la matriculación y la permanencia de estos niños en el contexto escolar (aula, patio, cancha deportiva y transporte escolar). Además, se incluyeron también aquellos relacionados con la estructura de los servicios de salud, como el ambulatorio de salud escolar y los servicios de salud en la escuela.

En cuanto a los tipos de estudios, se incluyeron artículos científicos con enfoques cuantitativos, cualitativos y métodos mixtos, además de estudios de casos de servicios de salud escolar para niños con TQT. En la búsqueda exhaustiva, se incluyó también la literatura gris, como tesis, disertaciones, programas de atención, directrices y editoriales. No se aplicó ningún límite de fecha o idioma a la investigación.

### Estrategia de Búsqueda y Selecc ión de los Estudios

En la metodología JBI^®^, la estrategia de búsqueda se implementa en tres etapas: 1) se realiza una búsqueda limitada a las bases de datos PubMed y CINAHL para identificar los términos más utilizados en los títulos y resúmenes de los artículos de interés para la pregunta de revisión; 2) los términos indexados se utilizan como ayuda en la elaboración y el perfeccionamiento de una estrategia de búsqueda sensible y específica; 3) se pone en práctica la estrategia de búsqueda completa, incluyendo los Descriptores en Ciencias de la Salud, *Medical Subject Headings*, sus respectivos sinónimos, palabras clave y *términos de entrada*. Esta estrategia final se adapta a cada fuente de información/base de datos. La estrategia de búsqueda aplicada a la biblioteca PubMed se ilustra en el Cuadro 1.

**Cuadro 1:** Ejemplo de una estrategia de búsqueda aplicada a la biblioteca PubMed (MEDLINE).

Fuente de información	Estrategia de búsqueda el 24 de julio de 2024
PubMed	((Tracheostomy[mh] OR Tracheostom*[tiab]) AND ("Infant"[mh] OR infant*[tiab] OR Child[mh] OR child*[tiab] OR Children[tiab] OR "Disabled Children"[tiab] OR "Children with Disabilities"[tiab] OR "Children with Disability"[tiab] OR "Disabled Child"[tiab] OR "Handicapped Children"[tiab] OR children special health care need*[tiab] OR "exceptional child"[tiab] OR "exceptional children"[tiab] OR "technology dependent child"[tiab] OR "technology dependent children"[tiab] OR "Special Needs Child"[tiab] OR "Special Needs Children"[tiab])) AND ("Mainstreaming, Education"[mh] OR Education Mainstreaming[tiab] OR Educational Mainstreaming[tiab] OR "Return to School"[mh] OR Return School*[tiab] OR "School Health Services"[mh] OR "School Health Services"[tiab] OR "School Based Health Services"[tiab] OR "School Based Services"[tiab] OR "School Health Promotion"[tiab] OR "School Health Service"[tiab] OR "School Based Health Service"[tiab] OR "School Based Health Services"[tiab] OR "School Based Service"[tiab] OR "School Based Services"[tiab] OR Middle School Teacher*[tiab] OR Elementary School Teacher*[tiab] OR "educational institution"[tiab] OR "school building"[tiab] OR "school organisation"[tiab] OR "school organization"[tiab] OR School*[tiab] OR "Elementary Education"[tiab] OR "Elementary School"[tiab] OR "Middle Schools"[tiab] OR "Primary Education"[tiab] OR "Secondary Education"[tiab] OR "intermediate school"[tiab] OR "junior high school"[tiab] OR "junior secondary school"[tiab] OR "lower secondary school"[tiab] OR "Schools"[mh] OR Primary School* OR Secondary School*[tiab] OR "Schools, Nursery"[mh] OR Nursery School*[tiab] OR "Students"[mh] OR Student*[tiab] OR "Education, Special"[mh] OR Special Education*[tiab] OR "Education"[mh] OR Education[tiab] OR Educational Activit*[tiab] OR Literacy Program*[tiab] OR "Teaching"[majr] OR Teaching[tiab] OR Educational[tiab] OR "Schools, Nursing"[mh] OR Nursing School*[tiab] OR Nurse Training School*[tiab] OR school aged[tiab])

Se revisaron las listas de referencias de todas las fuentes de evidencia incluidas para buscar estudios adicionales. No se impusieron restricciones en cuanto al idioma ni al periodo de tiempo. La última búsqueda se realizó el 24 de julio de 2024. Las bases de datos consultadas incluyeron PubMed, CINAHL (EBSCOhost), ERIC, PsycInfo, *Web of Science Core Collection*, Scopus/Portal de Periódicos de CAPES, SciELO, LILACS/Biblioteca Virtual en Salud, *Cochrane Database of Systematic Reviews* y *JBI Evidence Synthesis*. La búsqueda de estudios no publicados y literatura gris incluyó *OpenGrey*, Catálogo de Tesis y Disertaciones de CAPES, BASE, DART-*Europe*, Plataforma *Dimensions* y búsquedas manuales de listas de referencias de artículos de texto completo incluidos.

### Extracc ión y Análisis de Datos

Para la selección de las fuentes de evidencia, se reunieron los registros identificados y se cargaron en Mendeley™, eliminando los duplicados. Todo el proceso de selección de los estudios se llevó a cabo con la ayuda de la herramienta Rayyan por dos revisores independientes, evitando el riesgo de sesgo en la selección de los estudios de acuerdo con los criterios de inclusión para la revisión^(13)^. Cualquier desacuerdo que surgiera entre los revisores se resolvió mediante discusión o con un tercer revisor.

Para la extracción de los datos^(14)^, se utilizó una herramienta propia elaborada por los autores. Dos revisores, de forma independiente, extrajeron los datos de los resultados de los estudios seleccionados e introdujeron en la herramienta aquellos que respondían a las estrategias para facilitar la matriculación y la permanencia de los niños con TQT en clases escolares regulares. Tras esta etapa, los datos se presentaron a un tercer revisor, con *experiencia* en el método y el tema, que seleccionó los que mejor respondían a la pregunta de esta revisión de alcance para ser sometidos a análisis. En los casos en que hubo divergencias, los tres revisores se reunieron para llegar a un consenso.

Los principales hallazgos se presentan mediante un análisis de contenido descriptivo básico y se organizan en función de las preguntas de revisión de este estudio. Los datos extraídos se presentan en formato tabular y diagramático. Los resultados tabulados van acompañados de un resumen narrativo^(14)^.

## RESULTADOS

Se seleccionaron un total de 30 estudios para la extracción de datos y se incluyeron en la revisión. Los resultados de la búsqueda y del proceso de inclusión de los estudios se presentan de acuerdo con el diagrama de flujo PRISMA-ScR 2020 (Figura 1).

### Caracterización de los Estudios Incluidos

De los 30 estudios, 24 se realizaron en los Estados Unidos de América^(8,16–38)^, tres en Inglaterra^(9,39,40)^, dos en Japón^(10,41)^ y uno en Sudáfrica^(42)^ (Cuadro 2).

**Cuadro 2:** Caracterización de los estudios incluidos.

Autor, año	País Naturaleza/tipo de estudio	Naturaleza/tipo de estudio	Población/escenario	Objetivo del estudio
Mahomva et al.^(42) 2017^	Sudáfrica	Cualitativo	Cuatro madres, dos padres; cuatro profesores/Hogar y escuela	Recomendaciones para que las escuelas sean seguras y accesibles para los niños con T2D.
Kruger et al.^(16) 2009^	EE.UU. CUALITATIVO	Cualitativo	13 enfermeras/Escuela	Retos y beneficios en la interacción entre enfermeras, padres y personal en el cuidado de los niños.
Casey^(17) 2002^	EE.UU. CUALITATIVO	Cualitativo	18 enfermeras escolares/Escuela	Papel de la enfermera en salud escolar que trabaja con alumnos médicamente frágiles en un entorno escolar.
Mukherjee et al.^(9) 2000^	UK Cualitativo	Cualitativo	58 familiares, 34 profesores/escuela	Dificultades encontradas por los profesores en el cuidado de los niños.
Rehm and Rohr^(18) 2002^	EE.UU. CUALITATIVO	Cualitativo	11 familiares, nueve enfermeras, ocho profesores, cinco enfermeras escolares/Hogar, aula, enfermería	Cómo las familias, los educadores y los profesionales de salud garantizan la seguridad y la salud de los niños en el entorno escolar.
Shimizu and Katsuda^(10) 2015^	Japón	Cualitativo	11 profesores/escuela	Percepciones de los profesores sobre el papel de las enfermeras en la atención a los niños dependientes de la tecnología en las escuelas.
Shimizu and Suzuki^(41) 2015^	Japón Cualitativo	Cualitativo	21 enfermeras/escuela, aulas, sala de cuidados	Papel de las enfermeras en el cuidado de los niños dependientes de la tecnología en las escuelas.
Esperat et al.^(19) 1999^	EE.UU.	Método mixto	618 profesores, 43 enfermeras/escuela	Necesidad de educación y servicios sanitarios en el cuidado de NANEAS en las escuelas.
*American Academy Pediatrics: Committee on Injury and Poison Prevention* ^(20) 2001^	EE.UU.	Informe de directrices prácticas	NA	Directrices para niños con TQT durante el transporte.
Graff and Ault^(21) 1993^	EE.UU.	Guía práctica	NA	Directrices para el personal escolar que trabaja con alumnos con necesidades de salud especiales
Grundfast et al.^(8) 1988^	EE.UU.	Programa modelo de centro médico	NA	Integración de niños con TQT en el sistema escolar.
Haynie et al.^(22) 1989^	EE.UU.	Pautas de atención	NA	Directrices información útil para transformar los avances tecnológicos y el compromiso social de los niños.
Janz et al.^(23) 1993^	EE.UU.	Directrices de atención	NA	Discusión de los problemas de la inclusión de NANEAS en el aula ordinaria.
Lehr and Greene^(24) 2002^	EE.UU.	Pautas de práctica	NA	Cuestiones, problemas y prácticas para alumnos con necesidades de salud complejas en las escuelas.
Lehr^(25) 1990^	EE.UU.	Directrices prácticas	NA	Programas educativos para alumnos con necesidades de salud complejas.
Levine^(26) 1996^	EE.UU.	Estudio de caso	Cuatro alumnos y dos alumnas/Escuela	Experiencia de dependientes de soporte ventilatorio que asisten a la escuela.
Makrinioti et al.^(39) 2021^	Inglaterra	Editorial	NA	Un enfoque multidisciplinar para abordar las dificultades de la vuelta al colegio de los niños con TQT.
Margolis^(27) 2002^	EE.UU.	Legislación y políticas	NA	Utilización de leyes y políticas para los servicios de salud escolar.
Palfrey et al.^(28) 1992^	EE.UU.	Modelo de escuela consultiva	NA	Planificación y procedimientos de formación de habilidades.
Parette et al.^(29)^ 1994	EE.UU.	Modelo de inclusión	NA	Papel de la enfermera pediátrica.
Porter et al.^(30) 2013^	EE.UU.	Preparación para emergencias	NA	Instrucciones específicas de atención de emergencia para los alumnos en la escuela.
Raymond^(31) 2009^	EE.UU.	Estudio de caso	Niño de 6 años/Escuela	Proceso de integración, funciones del equipo e implicaciones para la práctica de la enfermería en salud escolar.
Smith and Leatherby^(32) 1992^	EE.UU.	Directrices	NA	Prestación de servicios a alumnos para procedimientos especiales de cuidados en salud en la escuela.
Toothaker and Cook^(33) 2018^	REVISIÓN NO SISTEMATIZADA USA	Revisión no sistematizada	NA	Habilidades de la enfermera en salud escolar para el cuidado de alumnos con TQT.
Mulligan-Ault et al.^(34) 1988^	EEUU CUANTITATIVO	Cuantitativo	150 profesores/escuela	El papel de la enfermera en salud escolar
Patel et al.^(35) 2009^	EE.UU.	Método mixto	23 niños/hospital	Experiencia de enfermera en salud escolar de niños con TQT.
Pufpaff et al.^(36) 2015^	EE.UU.	Cuantitativo	76 enfermeras escolares/Escuela	Colaboración entre la enfermera de salud escolar y el profesor.
Smith et al.^(40) 2003^	UK Cuantitativo	Cuantitativo	11 miembros de la familia y el cuidador del niño en la escuela/Hospital	Identificación de niños escolarizados y apoyo recibido.
Heller et al.^(37) 2000^	EE.UU.CUANTITATIVO	Cuantitativo	342 profesores, 49 profesionales de salud que no enfermeros, 21 enfermeras, diez administradores escolares/Escuela	Formación del personal en salud con habilidades para intervenir en el entorno seguro.
Sapien and Allen^(38) 2001^	EE.UU.CUANTITATIVO	Cuantitativo	230 enfermeras escolares/Escuela	Preparación de las escuelas de sistema público de educación para atender las necesidades de salud emergentes de acuerdo con las directrices.

Clave: EE.UU. – Estados Unidos de América. NA: no aplicable; TQT: traqueostomía; NANEAS: niños con necesidades de atención y cuidados en salud especiales.

Fonte: elaborada pelas autoras.

En cuanto a la naturaleza de los estudios, siete son cualitativos^(9,10,16–18,41,42)^, seis de ellos realizados mediante entrevistas individuales^(9,10,16–18,42)^ y un grupo focal^(41)^; cinco estudios cuantitativos^(34,36–38,40)^ con aplicación de cuestionarios; dos estudios de método mixto^(19,35)^; y 16 informes de directrices de práctica clínica^(20–25,32)^, informes de casos^(26,31)^, modelos consultivos^(8,28,29)^, editoriales^(39)^ y revisiones no sistemáticas de la literatura^(33)^. Los estudios cuantitativos^(34,36–38,40)^ incluyeron una población de 956 personas (enfermeras de salud escolar, enfermeras de atención primaria y hospitalaria, maestros de escuelas primarias, niños en edad escolar, padres). En los estudios cualitativos^(9,10,16–18,41,42)^ participaron 839 personas (madres, padres, cuidadores, enfermeras escolares, enfermeras y profesores), y se implementaron dos informes de casos^(26, 31)^ con siete niños. La estrategia de búsqueda recuperó documentos del periodo comprendido entre 1988 y 2021, siendo 11 estudios anteriores al año 2000^(8,19,21–26,29,30,33,35)^; y después de 2000, se contabilizaron 19 publicaciones^(9,10,16–18,20,24,27,28,30,31,33,35–39,41,42)^.

Las estrategias adoptadas por los profesionales de la salud, la educación y los cuidadores familiares para matricular y mantener la atención de los niños con TQT en escuelas regulares se organizan en tres modelos, como se muestra en la Figura 2.

**Figura 2:** Modelos de atención en salud escolar para niños con necesidades de salud especiales que viven con traqueotomía.

Fuente: Elaborado por las autoras.

### Modelo de Atención a la Salud Escolar Basado en la Colaboración Entre la Escuela y Los Servicios Sanitarios

El modelo de atención a la salud escolar basado en la escuela y los servicios de salud se centra en la colaboración entre los servicios de referencia para la continuidad del cuidado al niño, la escuela y los recursos de la comunidad. Juntos, planifican estrategias y acciones para garantizar la matriculación y la permanencia en el entorno escolar. Para ello, destacan: la implementación de recomendaciones de profesionales de la salud para atender las demandas específicas del niño en la escuela y proporcionar el equipo necesario; la identificación de situaciones de emergencia relacionadas con la TQT y formas de contacto con el equipo de emergencia (ambulancia o rescate o urgencias); la contratación de personal cualificado para el cuidado; la organización de una visita del servicio de salud a la escuela; y la previsión de una sesión de capacitación del personal escolar por parte del servicio de salud^(8,9,18,19,21–24,26,28,29,32,42)^.

Para ello, antes del inicio del año escolar, se prevé la participación de los profesionales de la salud en la gestión de emergencias en la escuela y la puesta a disposición de la comunidad escolar de información sobre el niño. Se da prioridad al desarrollo de políticas, protocolos y algoritmos, con la información esencial sobre las condiciones de salud del niño que debe anticiparse y comunicarse a la comunidad escolar. Se programa una reunión individual con el niño, los padres y los responsables en la escuela^(19,20,22,24–26,30,32,42)^.

El servicio de salud comparte con la escuela las especificidades del cuidado del niño con TQT, lo que puede constituir una emergencia y qué procedimientos deben realizarse en una intervención de emergencia. La atención se documenta debidamente en caso de que sea necesario recurrir a los servicios de salud de la comunidad. Con esta información, las escuelas adaptan su logística para atender las necesidades especiales de salud del niño, proporcionando un espacio fuera del aula cuando no se sienta bien, como una sala privada, climatizada, para la realización de procedimientos, con control de temperatura, acceso al agua para la limpieza del material y el lavado de manos, lugar para guardar el equipo y los suministros, enchufes eléctricos y fuentes alternativas de energía^(8,9,21–24,28,30,31)^.

Tan pronto como se completó la planificación de la transición para la matriculación del niño y se implementaron las medidas de adaptación ambiental, se iniciaron las estrategias para su permanencia en la escuela, con una evaluación después de un mes de inclusión escolar. Algunos estudios recomiendan, como parte de la política de inclusión escolar de niños con TQT, que la inserción segura requiere un profesional de enfermería disponible en la escuela, siempre que el niño lo necesite. Para garantizar su salud y seguridad, algunos médicos defienden que se acomode al niño cerca de la mesa del profesor, manteniendo una vigilancia atenta sobre su comportamiento. Es necesario estar alerta para informar a los padres sobre los riesgos potenciales de infección o los síntomas precoces, para que puedan mantener a los niños en casa o llevarlos a la atención médica tan pronto como se inicie una infección^(9,16–18,21,42)^.

Las escuelas cuentan con la colaboración de un profesional de la salud especializado para prescribir e indicar directrices estandarizadas para el cuidado de estos niños. Además, identifican a un miembro del equipo de salud como persona de enlace que, junto con los padres del niño, informa sobre su estado de salud y toma decisiones sobre el plan de cuidados. Se indica quién es responsable de la compra y el mantenimiento de los equipos, la eliminación de los residuos sanitarios y el suministro de los materiales necesarios para el cuidado del niño en la escuela^(8,9,20–22,26,40)^.

Para que el niño pueda seguir asistiendo a la escuela, el equipo de la comunidad escolar recibe formación del servicio de salud, que puede impartirse en la escuela o en el centro de salud de la comunidad. La capacitación es impartida por profesionales del cuidado domiciliaria, profesionales con experiencia clínica en pediatría, personas con formación y conocimientos sobre los derechos del niño (legislación), educadores previamente capacitados y familiares cuidadores con experiencia en procedimientos específicos realizados en sus propios hijos^(8,18,21–31,39,40)^. En este sentido, la capacitación es impartida por profesionales certificados para capacitar a maestros, administradores, personal auxiliar, empleados del transporte escolar, coordinadores, profesionales de enfermería en salud escolar (recién contratados) y compañeros de clase; es decir, todas las personas que mantienen contacto regular con el niño. Cuando el personal de la escuela está capacitado, los procedimientos específicos para el manejo de la TQT en el entorno escolar se llevan a cabo de manera segura y adecuada.

La capacitación se basa en competencias y habilidades para el manejo de problemas y se formaliza según las necesidades de los miembros de la comunidad escolar y de los cuidadores que prestan atención directa a los niños en la escuela. Se utilizan recursos gráficos (videos), sesiones de enseñanza, preguntas y respuestas, información verbal adicional de las familias, resolución de problemas y visitas periódicas con sesiones de actualización para ofrecer apoyo y mantener sus habilidades. La escuela y el servicio de salud documentan y refuerzan la capacitación de vez en cuando, capacitando al menos a dos o tres personas para que haya apoyo disponible^(4,8,10,11,13,14,18,20,25,27,29,31,39,40)^.

En resumen, las pruebas cualitativas señalan como temas tratados en la formación las normas sobre quién puede realizar procedimientos especializados y complejos en el aula, el acceso a los recursos comunitarios y las formas de contacto en casos de urgencia. Se incluyeron conocimientos básicos sobre la TQT aplicados al contexto escolar, plan de emergencia, signos de malestar, estado respiratorio y general del niño para atender necesidades de salud especiales.

Las evidencias cuantitativas indicadas en la Tabla 1 destacan los temas reanimación cardiopulmonar (RCP), primeros auxilios y cuidados generales con la TQT. La necesidad de capacitación incluye procedimientos de aspiración de las vías respiratorias mediante TQT, uso de dispositivos y administración de oxígeno^(8,18,21–25,27–29,31,32,42)^.

**Tabla 1:** Temas tratados en las sesiones de formación para el personal escolar, bajo la responsabilidad del servicio de salud, la enfermería de salud escolar y la propia escuela, como estrategia para mantener a los niños con traqueostomía en la escuela^(34,35,37,38,40)^,1988–2015.

Topics covered in training	Profesionales sanitarios^(37,38)^ (n = 572)			Familiares y cuidadores no profesionales (n = 643)		
	Profesional de enfermería en salud escolar (n = 1.078)	Médico (n = 88)	Profesionales de la rehabilitación (n = 132)	Servicios sanitarios (n = 159)	Familia (n = 444)	Cuidador (profesor + mediador) (n = 199)
Primeros auxilios^(37)^		256		–	–	–
Reanimación cardiopulmonar^(37,38)^		526		–	–	–
Aspiración^(37)^ n = 559	283	24	38	48	113	53
Cuidados generales de traqueotomía^(37)^ n = 539	272	17	30	49	128	43
Uso de inhalador/nebulizador^(37)^ n = 516	250	23	23	28	127	65
Administración de oxígeno^(37)^ n = 486	273	24	41	34	76	38

Fuente: Elaboración propia.

En la Tabla 1, los profesionales de la salud (enfermeros de salud escolar, médicos y profesionales de la rehabilitación), familiares y cuidadores no profesionales de la salud (profesores y mediadores capacitados), que están capacitados, pueden entrenar al resto de los miembros del equipo escolar. Los temas más recurrentes en la formación en la escuela son la RCP y los primeros auxilios (782 casos), seguidos de la aspiración de las vías respiratorias mediante TQT (559). Sin embargo, solo los profesionales de la salud impartieron formación sobre primeros auxilios y RCP. Los demás temas, como los cuidados generales con la TQT, el uso de equipos (inhalador y nebulizador) y la administración de oxígeno, fueron implementados por familiares con experiencia o cuidadores previamente capacitados.

### Modelo de Atención Basado en el Servicio de Enfermería de Salud Escolar

En este modelo, el profesional de enfermería em salud escolar forma parte del equipo educativo de la escuela y asume la función de coordinador de salud infantil en la escuela, conectando a la familia, la comunidad, los profesionales sanitarios y los educadores. Como miembro del servicio de salud escolar, es responsable de la prestación de cuidados directos al niño con TQT durante su permanencia en la escuela. Se mantiene capacitado (capacitaciones), informado y actualizado sobre las evidencias más recientes relacionadas con el cuidado de estos niños. Cuenta con el apoyo del maestro de aula para proporcionar la comunicación y la información esenciales para evaluar la salud y el bienestar del niño (socialización con otros niños); asiste a las reuniones de planificación educativa; y cuenta con el apoyo del director de la escuela para revisar la planificación de salud en la escuela recomendada para este equipo^(10,16,17,22,31,33,41)^.

El profesional de enfermería en salud escolar mantiene un canal de comunicación con los médicos de referencia del niño, con los servicios de salud de la comunidad o con otros profesionales que acompañan la salud del niño, informándose sobre las condiciones del niño. Es un profesional cualificado para realizar cuidados respiratorios en la aspiración traqueal, la evaluación diaria de la piel periestomal, la observación de signos de complicaciones (sangrado o infección) y el cambio de la cánula de TQT siempre que sea necesario. Estas demandas de cuidados se integran en los cuidados diarios, ajustándolos al horario escolar, por ejemplo, a la entrada y salida de la escuela, al bajar y subir del autobús escolar^(16–18,33,41)^.

De esta manera, este modelo reúne un conjunto de estrategias que facilitan la matriculación del niño y su permanencia en la escuela, cuando llega el momento de la transición del hogar a este entorno. Entre estas estrategias, destacan el establecimiento de las atribuciones del profesional de enfermería en salud escolar, la planificación de visitas domiciliarias para conocer la rutina diaria y mantener la continuidad de los cuidados, y la simulación de diferentes escenarios de emergencia con el equipo escolar (profesores, directivos, personal de apoyo, brigadista). Además, informa al equipo de la escuela y al servicio de transporte escolar sobre las necesidades del niño, como es el caso del mantenimiento de la humedad en ambientes climatizados (aire acondicionado o calefacción), y presenta un plan de acción para las actividades realizadas fuera de la escuela (excursiones)^(16,17,20,22,26,30,31,41)^.

En cuanto a los cuidados directos, es un profesional con competencias y habilidades en el manejo de la TQT y en la evaluación diaria del estado del niño y del estoma, incluyendo el cambio de la cánula, revisando y ejecutando protocolos de emergencia con medidas de rescate para garantizar un entorno seguro y saludable en el entorno escolar público^(16,17,22,33)^.

En este modelo, la relación del profesional de enfermería con la familia consiste en la creación de un grupo de apoyo parental como una oportunidad para que los cuidadores se apoyen mutuamente. Se informa sobre las condiciones del niño con los padres, verifica la necesidad de capacitación de los miembros de la comunidad escolar sobre situaciones de emergencia, realizando capacitación con familiares, maestros y personal escolar en la realización de procedimientos como RCP, cuidados con la TQT y auscultación pulmonar. Se encarga de la capacitación y la delegación de tareas, la supervisión del desempeño en la realización de procedimientos y el cuidado de ese niño por parte del personal de la comunidad escolar. La delegación de tareas se basa en la competencia inicial y continua de los miembros del equipo escolar para garantizar una atención segura y eficaz^(16–17,19,22.27,32)^.

Esta formación puede ser impartida por personal de la propia escuela (a quien se le delegará la tarea del cuidado), que será formado por profesionales de la salud (enfermeiros de salud escolar), quienes también actuarán como difusores de conocimientos y habilidades para otros miembros del equipo escolar. En aquellas escuelas que cuentan con servicios de enfermería en salud escolar, el modelo de atención se basa en el profesional de enfermería de salud escolar, y la capacitación es esencial para garantizar condiciones seguras para la permanencia de estos niños en la escuela. Sin embargo, es necesario que los profesionales de la educación que integran el equipo escolar reciban formación y tengan acceso a fuentes de información fiables sobre los cuidados complejos que requiere este niño (Tablas 1 y 2). Las escuelas que desarrollan programas de formación del equipo escolar abordan temas relacionados con los procedimientos de aspiración, el cambio de la cánula de la TQT, la RCP y los cuidados generales del niño^(35,40)^.

**Tabla 2:** Formación de profesionales de la educación y fuentes de información sobre el cuidado de niños con traqueostomía en la escuela como estrategia para mantener la asistencia escolar^(25–30)^, 1988–2015.

Variables	N (384)	%
**Profesionales de la educación formados**		
Personal docente	150	39
Programa formal	76	20
Informal	64	17
Con la familia	54	14
En un programa desarrollado por una universidad	40	10
**Fuentes de información sobre las necesidades y demandas de atención infantil**	**N (247)**	**%**
Familia	83	34
Médico de familia	58	23
Enfermera en salud escolar	58	23
Profesionales de la rehabilitación	48	20

Fuente: Elaboración propia.

### Modelo de Atención en Salud Escolar Basado en la Familia Basado en la Familia

En este modelo de atención a la familia, se inicia el proceso de matriculación del niño con TQT en la escuela, comunicando claramente la información sobre sus necesidades especiales de salud y de servicios al personal escolar (profesores, directores y personal de apoyo). El equipo trabaja de manera colaborativa para compartir información con los futuros compañeros de clase del niño y participa activamente en el proceso de integración, enviando cartas y fotos antes del inicio de las clases y en las reuniones con el director y el maestro. Como parte interesada en la seguridad del niño en la escuela, contribuye a la planificación de su permanencia, proponiendo una formación para la comunidad escolar, ofreciendo recomendaciones específicas para las adaptaciones y la logística del entorno escolar e informando sobre las necesidades de salud que requieren un plan de emergencia. Los familiares cuidadores expresan a la dirección de la escuela la necesidad de contratar profesionales de enfermería (de salud escolar) para capacitar, monitorear y supervisar al personal escolar en las intervenciones de salud que el niño necesita^(9,17,31,32)^.

La familia mantiene una comunicación frecuente con el profesional de enfermería de salud escolar, cuando este está presente en la escuela. Al conocer todo el historial y las condiciones de salud del niño, se convierte en socio en el proceso de toma de decisiones, participando en la elaboración de un plan de emergencia, documentado y por escrito, que se distribuye entre las personas que están en contacto con el niño en la escuela. Este plan incluye llamar a los padres en caso de emergencia y decidir cuándo es apropiado llevar al niño al hospital y/o a los servicios de salud de la comunidad. Además, son responsables de proporcionar el equipo y los suministros necesarios para continuar el cuidado del niño mientras está en la escuela^(9,17,31,32)^.

En este modelo de atención, los padres del niño con TQT actúan como facilitadores para garantizar el derecho a la matriculación y la permanencia de este niño en la escuela. Por ejemplo, el familiar permanece en el centro escolar y visita el aula dos o tres veces para supervisar el estado del niño durante su estancia en la escuela hasta que sea capaz de autocuidarse en el manejo de la TQT, la limpieza de las vías respiratorias, el control de la tos y la eliminación de secreciones^(18,42)^.

## DISCUSIÓN

Los tres modelos de atención en salud escolar descritos en esta revisión apuntan a una inclusión escolar basada en las necesidades de salud especiales y en la naturaleza compleja de los cuidados continuos y prolongados que requieren los niños con TQT fuera del entorno doméstico. Los resultados indican el papel fundamental de los familiares, los servicios sanitarios y la escuela, que deben actuar de forma colaborativa y organizada para garantizar la matriculación y la permanencia en la escuela.

Cada modelo de atención desarrolló estrategias de cuidados pensadas conjuntamente con la familia^(9,18,42)^, con la presencia de enfermería de salud escolar^(17,19,34,36,38)^ y basadas en la escuela y el servicio de salud^(16,22,30,32,39)^. Los tres modelos de atención en salud escolar presentaron estrategias facilitadoras para la matriculación y la permanencia de los niños con TQT en la escuela. Además, los modelos demuestran la capacidad de proporcionar cuidados específicos al niño en la escuela y de establecer y ejecutar un plan de emergencia de acuerdo con las directrices de emergencias respiratorias para niños con TQT. Se prevé la necesidad de contar con personal cualificado, formado y capacitado para actuar de inmediato, evitando daños a la integridad física del niño.

Los estudios elegibles que respondieron a la pregunta de la revisión proceden, en su mayoría, de contextos internacionales. Los países anglosajones (Estados Unidos de América, Reino Unido e Inglaterra) representan la mayoría de los estudios, seguidos por Japón, es decir, países con un mayor nivel de desarrollo económico que cuentan con servicios de salud escolar como política institucional. En el estudio de Sudáfrica, país con menor nivel de desarrollo económico, las evidencias apuntan a la necesidad de mantener escuelas más seguras para los niños con TQT.

Cabe destacar que entre los estudios recuperados no hay ninguno de origen brasileño. Desde la Constitución Federal de 1988 de Brasil, los derechos del niño están previstos en el artículo 227, siendo «deber de la familia, la sociedad y el Estado garantizar al niño y al adolescente, con absoluta prioridad, el derecho a la vida, a la salud, a la alimentación, a la educación, al esparcimiento, además de protegerlos de toda forma de negligencia, discriminación y opresión»^(43)^.

Los avances en la garantía de los derechos del niño se consolidan con la promulgación de la Ley Federal n.º 8.069, de 1990, del Estatuto del Niño y del Adolescente^(44)^, que dispone la protección integral del niño y del adolescente, que gozan de todos los derechos fundamentales inherentes a la persona humana, sin perjuicio de la protección integral. Se les garantizan oportunidades y facilidades que les permiten el desarrollo físico, mental, moral, espiritual y social, en condiciones de libertad y dignidad.

Sin embargo, el mapeo de las evidencias apunta a la necesidad de implementar un modelo de atención que favorezca la inclusión escolar de los niños con TQT. De esta manera, se respetan sus derechos de ciudadanía, evitando que se violen en caso de que no se satisfagan sus necesidades de movilidad en el espacio urbano (transporte escolar adaptado), sus necesidades de asistencia social en la provisión de condiciones materiales de existencia (insumos para la logística del cuidado continuo) y en la facilitación de su inclusión en la comunidad y la convivencia social.

Según la Constitución Federal de 1988^(43)^ y el Estatuto del Niño y del Adolescente^(44)^, todo niño tiene derecho a asistir a la escuela. Es nuestra responsabilidad social y política, como sociedad civil, desarrollar una conciencia crítica y exigir a las autoridades el cumplimiento de las directrices legales que protegen el interés superior del niño. Además, debemos recordar que las acciones individuales no sustituyen las tareas del Estado, pero las acciones colectivas movilizan a los agentes públicos y organizan mejor el proceso de supervisión del cumplimiento o incumplimiento por parte del Estado de sus deberes constitucionales, lo que nos llevará a buen término en la lucha contra el problema de la inclusión escolar de los niños con necesidades de salude especiales.

En Brasil, la Política Nacional de Atención Integral a la Salud de la Niñez^(45)^ articula un conjunto de estrategias intra e intersectoriales para la inclusión de estos niños en las redes temáticas de atención a la salud, mediante la identificación de vulnerabilidades y riesgos de agravamientos y enfermedades, reconociendo las especificidades de este público para una atención resolutiva. Existen lagunas y pocos programas de atención dirigidos a las necesidades específicas de los niños con cuidados asociados a la TQT, por ejemplo. La política no garantiza una gestión de los cuidados orientada a las necesidades de estos niños que necesitan ir a la escuela. Es necesario un enfoque inclusivo que apunte a estrategias y resoluciones para garantizar el acceso a los servicios de rehabilitación, transporte y educación para este grupo infantil.

Especialmente, al tratarse de niños con mayor riesgo de complicaciones, como la obstrucción de la cánula o la decanulación, estas pueden evitarse con la atención adecuada prestada por personal capacitado en RCP y primeros auxilios^(4,5)^. De este modo, se evitan consecuencias catastróficas cuando estos eventos ocurren en la escuela. La Ley Lucas^(46)^ (Ley n.º 13.722, de 4 de octubre de 2018) hace obligatoria la formación en primeros auxilios básicos para los profesores y el personal de los centros de enseñanza pública y privada de educación básica, señalando la necesidad que tiene el entorno educativo de hacer frente a las urgencias médicas de cualquier niño. En este sentido, esta ley respalda al personal de la escuela para actuar en emergencias, siempre que esté debidamente capacitado.

En Brasil, el Programa de Salud Escolar (PSE)^(47)^ es una iniciativa intersectorial de salud y educación^(46)^ instituida por el Decreto Presidencial n.º 6.286 de 2007 para contribuir a la formación integral de los estudiantes de las redes públicas de educación básica, fundamental y media. Se trata de una estrategia implementada por el equipo de salud familiar de la red de atención básica del Sistema Único de Salud, ejecutada con carácter consultivo a través de visitas programadas a la escuela. Sus acciones tienen como objetivo la detección precoz de hipertensión arterial, déficits visuales y auditivos, problemas de salud desatendidos y trastornos psicosociales.

El PSE se asemeja al modelo basado en la escuela y el servicio de salud, pero con limitaciones para satisfacer las necesidades de los niños con TQT, debido a su complejidad clínica, el riesgo de emergencia respiratoria y la protección de la integridad física del niño en la escuela.

Tanto la Constitución Federal como la Ley de Directrices y Bases de la Educación Nacional^(7)^ prevén el derecho a la educación, es decir, la matriculación y la permanencia del niño en la escuela. Sin embargo, la inclusión escolar implica la necesidad de que la normativa se plasme en políticas, programas y en un cambio cultural de la sociedad. Para ello, es necesario promover la reflexión sobre las necesidades de adaptaciones estructurales y sociales para acoger a estos CRIANES.

La inclusión escolar nos hace reflexionar que, a pesar de todas las limitaciones y barreras que los niños con TQT pueden enfrentar en la escuela, este será el lugar que los convertirá en sujetos activos y libres en su propia realidad. La escuela se presenta como un lugar privilegiado para la liberación, ya que solo a través de la producción de debates, discusiones y diálogos se puede alcanzar la comprensión de la realidad y, así, promover transformaciones en la sociedad^(48)^.

En Brasil, las clases domiciliarias se han mostrado una alternativa para las familias que mantienen el proceso de escolarización de los niños con TQT en edad obligatoria de asistir a la escuela^(1,7)^. Otro aspecto es el hecho de que el PSE se centra en la promoción de la salud, la prevención de enfermedades y el desarrollo de acciones que evalúan la situación vacunal, nutricional, bucodental y ocular, realizadas por equipos de salud familiar, según la demanda de la escuela y las acciones específicas definidas por la gestión de salud en el área de atención^(47)^. Por lo tanto, las acciones que se desarrollan no cubren las necesidades de los niños que viven con TQT, lo que representa un reto para la escuela, las familias y los propios niños.

## CONCLUSIÓN

Las estrategias que facilitan la matriculación y la permanencia en la escuela revelaron características distintas que se clasificaron en tres modelos de atención en salud escolar: basado en la familia, en la intersectorialidad salud-educación y en el ambulatorio de salud escolar. Cada uno de ellos presenta especificidades para atender las demandas de atención a los niños con TQT, identificando personal habilitado, formado y capacitado para intervenir con seguridad. Corresponde a las instituciones escolares y de salud, en colaboración con la familia, establecer acciones y estrategias, planificar con anticipación la matriculación y garantizar condiciones para la permanencia segura en la escuela, con una definición clara de roles y responsabilidades, además de definir planes de emergencia y protocolos adecuados para el mantenimiento de cuidados continuos y complejos en la escuela.

Esta revisión señaló la escasez de estudios sobre las estrategias de atención a los niños con TQT en países de bajos ingresos, especialmente en América del Sur, donde la colaboración entre los sectores de salud y educación aún es incipiente. Para aumentar la calidad y la relevancia de los resultados de la investigación, es fundamental realizar más investigaciones sobre la inclusión escolar de los niños con TQT en países de bajos y medianos ingresos. La documentación continua de las acciones y estrategias dirigidas a los niños con TQT en las escuelas puede contribuir a poner de relieve los casos de éxito de los que pueden beneficiarse otros contextos.

Es importante destacar que la revisión del alcance identificó escasas prácticas registradas en países de bajos ingresos, que se enfrentan a retos en la aplicación de la legislación y las políticas dirigidas a los CRIANES. Las posibles barreras para la inclusión de los niños con TQT en las escuelas incluyen la falta de planificación de las acciones de seguimiento fuera del hospital, los recursos limitados para hacer frente a la complejidad de la salud de estos niños, las deficiencias en la competencia técnica y la logística para atenderlos en la escuela, así como la falta de coordinación entre la escuela y los servicios de salud para garantizar la matriculación y la permanencia en la institución. Las evidencias presentadas ofrecen información valiosa para facilitar la inclusión de los niños con TQT en la escuela, contribuyendo a la construcción de un modelo alineado con el contexto. Para garantizar el derecho de acceso de los niños con TQT a la inclusión escolar y a la convivencia con sus compañeros, es necesario que se realicen futuras investigaciones para llenar las lagunas sobre las estrategias intersectoriales de salud y educación dirigidas a las especificidades del cuidado continua de este grupo de niños y jóvenes en las escuelas.

## Data Availability

Los datos de esta revisión de alcance están disponibles en la versión en portugués de la tesis doctoral de autoría de la primera autora. Base Minerva UFRJ. Enlace de acceso: https://minerva.ufrj.br/F/VVBF21NFGLQT47PQI1PD9LV29HH6KSR7QXLPLJXXDU27C6ELAH-04989?func=service&doc_library=UFR01&doc_number=000958274&line_number=0001&func_code=WEB-BRIEF&service_type=MEDIA.
